# A *de novo* dual-targeting supramolecular self-assembly peptide against pulmonary metastasis of melanoma

**DOI:** 10.7150/thno.83819

**Published:** 2023-06-26

**Authors:** Jingjing Wang, Xiaoqiang Zheng, Xiao Fu, Aimin Jiang, Yu Yao, Wangxiao He

**Affiliations:** 1 Department of Medical Oncology, The First Affiliated Hospital of Xi'an Jiaotong University, Xi'an 710061, China.; 2 Institute for Stem Cell & Regenerative Medicine, The Second Affiliated Hospital of Xi'an Jiaotong University, Xi'an 710004, China.; 3 Department of Talent Highland, The First Affiliated Hospital of Xi'an Jiao Tong University, Xi'an 710061, China.

**Keywords:** Peptide, Wnt/β-catenin, Supramolecular self-assembly, Melanoma metastasis, Cancer therapy

## Abstract

Despite recent advances in treatment, overall survival rates for metastatic melanoma, especially those that invade the lungs, continue to be low, with 5-year survival rates of only 3% to 5%. It was recently discovered that Wnt/β-catenin signaling pathways and MAPK/ERK signaling pathways are involved in melanoma metastasis.

**Methods:** Herein, a bifunctional supramolecular peptide termed HBB^plus^@CA was constructed by a self-assembling RGD-modified MAPK/ERK peptide inhibitor (HBB^plus^) and a small molecule catenin inhibitor (carnosic acid (CA)).

**Results:** Expectedly, the HBB^plus^@CA could internalize melanoma cells, accumulate in the tumor-bearing lung, and be biosafe. As designed, HBB^plus^@CA simultaneously suppressed both Wnt/β-catenin and MAPK/ERK signaling pathways and suppressed melanoma cell proliferation, migration, and invasion in more action than CA or HBB^plus^ monotherapy. More importantly, HBB^plus^@CA demonstrated potent inhibition of lung metastasis in mice bearing metastatic melanoma of B16F10 and significantly prolonged their survival.

**Conclusion:** In summary, a supramolecular peptide-based strategy was not only developed to suppress pulmonary metastasis of melanoma, but it also renewed efforts to identify cocktail drugs that act on intracellular targets in various human diseases, including cancer.

## Introduction

Metastatic melanoma has a poor prognosis, particularly lung metastatic cancer, with only a 6- to 9-month median survival rate and a 5-year survival rate of only 3% to 5% 1, 2. Several novel treatment options have improved survival times for patients with metastatic melanoma, including immune checkpoint inhibitors that have increased 5-year overall survival (OS) rates from 15% to 30% 3-5. However, therapy resistance and recurrence are common occurrences 6-8. Hence, it is of extreme importance to improve current therapeutic strategies, suppress melanoma metastasis, and prolong survival for patients suffering from melanoma.

**Figure 1:**
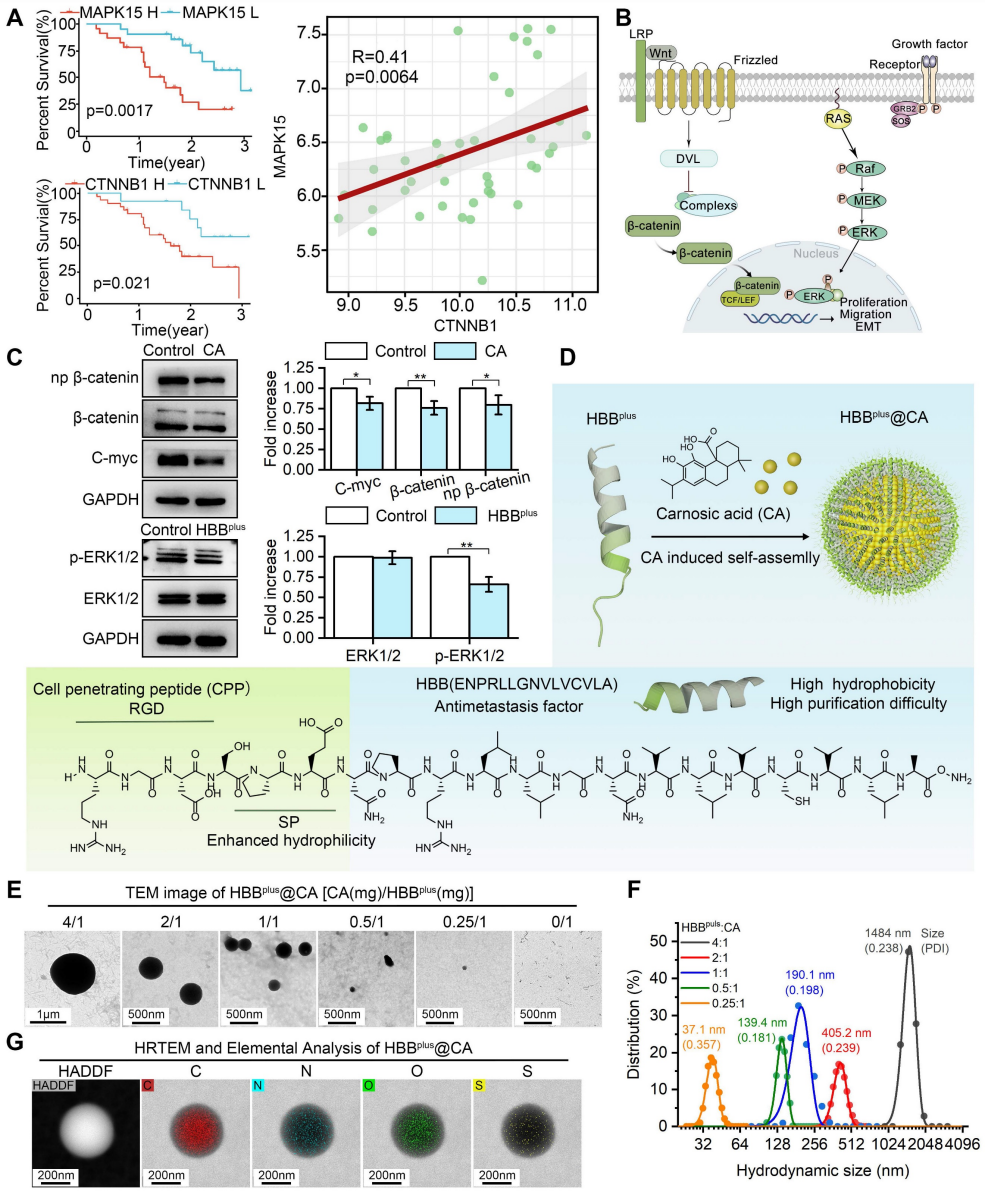
** Design and Preparation of the Supramolecular Peptide HBB**^plus^**@CA.** (A) Bioinformatics analysis of the function of Wnt/β-catenin and MAPK/ERK signaling pathways in metastatic melanoma. In the GSE30219 cohort, Univariate Cox regression analysis of CTNNB1 and MAPK15 for metastatic melanoma. CTNNB1 was positively associated with MAPK15. (B) Wnt/β-catenin signaling pathways and MAPK/ERK signaling pathways promote the tumor metastasis process. (C) Western blot analysis for the expressions of β-catenin, np-β-catenin, and c-Myc proteins after CA treatment or the terms of ERK1/2/p-ERK1/2 after HBB^plus^ treatment in B16F10 cells. (D) Synthesis and schematic of HBB^plus^@CA particles. (E) TEM image of HBB^plus^-assembled nanoclusters induced by different concentrations of CA. (F) Size distribution of HBB^plus^-assembled nanoclusters induced by different concentrations of CA, measured by DLS. (G) HRTEM and elemental analysis by electron micrographs of HBB^plus^@CA formed by self-assembly of CA and peptides in a 1:1 ratio configuration. The data were presented as mean ± s.d. *, p < 0.05; **, p < 0.01; ***, p < 0.001.

Several recent studies have pointed out that the Wnt/β-catenin and MAPK signaling pathways are critical contributors to the metastasis of melanoma 9-15. The over-accumulation of β-catenin promotes the development of melanoma and melanoma lung metastases through activation of the classical Wnt pathway, and it has been confirmed that inhibiting Wnt/β-catenin can reduce the probability of melanoma lung metastase 9, 11, 16-18. Besides, a hyperactivated MAPK pathway may also contribute to melanoma metastasis through phosphorylation of ERK1/2 13, 19, 20, and it has been demonstrated that up to 90% of melanomas are chronically activated by this mechanism 21, 22. Furthermore, the present study examined clinical data cohorts from Gene Expression Omnibus (GEO) that provide detailed information on metastatic melanoma prognosis and gene expression and found a potential interaction between Wnt and MAPK signaling pathways on melanoma metastasis (Figure 1A). Therefore, suppressing both the Wnt and MAPK signaling pathways may significantly reduce the development of melanoma metastases in the lungs.

Several drug discoveries have been conducted in recent years to investigate the aberrantly activated Wnt/β-catenin and MAPK pathways 23-27. The compound carnosic acid (CA) has been suggested as a potential therapeutic agent that suppresses the nuclear translocation of oncogenic β-catenin 18, 28-30. Although it is a potent inhibitor of the Wnt/β-catenin pathway, its high hydrophobicity prevents it from being clinically beneficial 31. Besides, a peptide motif (METOX, sequence: ENFRLLGNVLVCVLA) in the C-terminal of human hemoglobin subunits (HBB2/HBB) inhibits the MAPK pathway by reducing ERK phosphorylation, thereby inhibiting tumor lung metastases 32. Although METOX had extraordinary potency* in vitro*, it was also compromised by inherent defects of peptides, such as high immune-phagocytosis and poor tumor targeting, which severely limited its anti-tumor efficacy *in vivo*.

To overcome CA's hydrophobicity and METOX's inherent defects, we herein developed a self-assembly strategy to combine CA and METOX into a supramolecular nanomedicine with enhanced tumor targeting and weakened side effects 33. First, RGDSP-modified METOX (HBB^plus^) as an amphiphilic anticancer peptide (ACP) increased the permeability to tumor cells, targeting and anticancer activity of HBB 34-36. Then, under hydrophobic and hydrogen bonding interaction, CA and HBB^plus^ self-assembled into a nanosphere and bifunctional supramolecular peptide termed HBB^plus^@CA 37-43. Using biophysical analysis, we determined that HBB^plus^@CA could penetrate cell membranes effectively in melanoma cells and prefer accumulation in tumor-bearing lung tissue. As intended, HBB^plus^@CA *in vitro* and *in vivo* inhibited both Wnt/β-catenin and MAPK/ERK signaling pathways simultaneously. Moreover, HBB^plus^@CA suppresses pulmonary metastases of B16F10 melanoma in C57BL/6 mice while exhibiting favorable safety data. Collectively, this study has demonstrated that supramolecular peptide-based strategies can suppress the development of pulmonary metastasis of melanoma, as well as inspire efforts to discover cocktail drugs targeting intracellular targets in a wide variety of human diseases.

## Results and Discussion

### Wnt/β-catenin and MAPK signaling pathways are involved in melanoma metastasis

To analyze the function of Wnt/β-catenin and MAPK/ERK pathways in metastatic Melanoma, two clinical data cohorts (GSE19234 and GSE30219) that provide detailed information on metastatic melanoma prognosis and gene expression were downloaded from GEO. First, we conducted a Kaplan-Meier survival curve analysis based on a log-rank test to identify prognostic factors derived from CTNNB1 (gene name for β-catenin) or MAPK15 (a representative gene in the MAPK family) for metastatic melanoma. In contrast to metastatic melanoma patients with low-CTNNB1, patients with high-CTNNB1 had a shorter OS (Figure 1A). As well, patients with high levels of MAPK15 had shorter OS compared to patients with low levels of MAPK15 (Figure 1A). Moreover, a correlation analysis was conducted on the mRNA levels of CTNNB1 and MAPK15, showing a significant positive correlation with a high coefficient of 0.41 (Figure 1A). The above results are consistent with the idea that the Wnt/β-catenin and MAPK/ERK signaling pathways are responsible for promoting the metastasis process in melanoma (Figure 1B). Therefore, simultaneous targeted inhibition of the Wnt-β-catenin and MAPK/ERK signaling pathways against metastatic melanoma is a feasible therapeutic strategy. Next, we tested the core-related protein expressions of B16F10, a metastatic melanoma cell line, after treatment of CA or HBB^plus^. As shown in Figure 1C and S1A, CA significantly downregulated β-catenin expression and its downstream protein c-Myc expression, suggesting the suppression of Wnt/β-catenin signaling pathways. Further, the HBB^plus^ peptide reduced the level of phosphorylated ERK, an important MAPK activator (Figure 1C and S1B).

### Design and construction of the supramolecular peptide HBB^plus^@CA

To increase cellular internalization and tumor targeting, an RGD-derived motif (RGDSP) was conjugated N-terminally to the METOX peptide resulting in the fusion peptide named HBB^plus^ (sequence: RGDSPENPRLLGNVLVCVLA). As depicted in Figure S2, ESI-MS measurement revealed that the molecular weight of HBB^plus^ was 2122.44 Da. The chemical formula of HBB^plus^ in Figure 1D indicated an amphiphilic peptide with an amino-terminal hydrophilic fragment and a carboxyl-terminal hydrophobic fragment. Therefore, the HBB^plus^ should be able to self-assemble with the hydrophobic CA according to the similarity principle 14, 18, 37, 38, 43-46.

The hydrophobic tails of HBB^plus^ interact and gather together, forming a hydrophobic core. At the same time, the hydrophilic head interacts with water molecules to wrap around the core, forming a stable micelle structure. The hydrophobic drug CA will be encapsulated in the hydrophobic area of the micelles during micellar assembly. CA is immobilized inside the micelles by interacting with the hydrophobic tails of the micelles (hydrophobic interactions and hydrogen bonding) (Figure 1D). We used the GROMACS 2018.3 software package and combined it with the MARTINI coarse-grained model (version 2.2) to conduct dynamics simulations on a system composed of 24× HBB^plus^ peptide molecules and 100× CA molecules in an aqueous solution during 5000 ns. As shown in Figure S3, CA, and HBB^plus^ peptides could self-assemble into the nanosphere. To verify it, CA was mixed with HBB^plus^ in mass ratios ranging from 4:1 to 0.25:1. The analysis of transmission electron microscopy (TEM) revealed that CA can trigger the self-assembly of HBB^plus^ into nanospheres, whereas HBB^plus^ alone is incapable of undergoing such a process (Figure 1E). Furthermore, there was a strong positive correlation between the concentration of CA and particle size (Figure 1E). According to dynamic light scattering (DLS) measurements, the hydrodynamic size of HBB^plus^@CA at a ratio of 1:1 was 190.1 nm with a polydispersity index (PDI) of 0.198 (Figure 1F), which was suitable for enhanced permeability and retention (EPR) effects 46-50, and thus intended to be a fixed ratio for the production of HBB^plus^@CA.

According to Figure 1G, high-resolution electron microscopy (HRTEM) images overlayed with elemental analysis of HBB^plus^@CA nanoparticles reveal a uniform distribution of C, N, O, and S within the particles, indicating uniform self-assembly. HBB^plus^@CA, CA, and HBB^plus^ were then subjected to Fourier transform infrared spectroscopy (FT-IR), which cannot only study molecule structure and chemical bonding but also characterize and identify chemical species. According to the FT-IR spectrum of HBB^plus^@CA, the strong absorption peak at 1000 cm^-1^ suggested the ionization or hydrogen bonding interaction of the carboxyl group of CA; the disappearance of the amide II band (1550-1650 cm^-1^) indicated that CA induced hydrogen bond rearrangement in the peptide; the broadening and dulling of the amino group absorption peak suggested the formation of hydrogen bonds between the free amino groups in the peptide and the carboxyl groups of CA. Additionally, the addition of CA likely led to conformational changes in the peptide, as evidenced by the enhanced C-H stretching vibration at 2950 cm^-1^. These findings suggested that CA interacted with the peptide and induced conformational changes in the peptide, indicating that the supramolecular peptide was successfully constructed (Figure S4). In the UV spectrum, the surface plasmon resonance effect and the local electric field effect accompanied by the HBB^plus^@CA nanostructure led to a red shift of the CA absorption peak (from 286.4 nm to 283.4 nm). Additionally, the formation of the nanostructures triggered solvent effects, leading to an elevated baseline of HBB^plus^@CA. These findings confirmed the formation of CA-induced nanoparticles (Figure S5). Besides, we conducted nuclear magnetic resonance (NMR) hydrogen spectroscopy measurements on CA and HBB^plus^ and integrated the chemical shifts of the hydrogen peaks. The hydrogen spectrum peaks corresponding to different functional groups of CA were shown in Figure S6A. Due to the molar ratio of CA to HBB^plus^ 10:1, the hydrogen spectrum peaks of the peptide in HBB^plus^@CA were very weak (marked in purple in Figure S6B). However, these changes in the hydrogen spectrum also provided helpful information: the hydrogen spectrum peak of the hydroxyl group in HBB^plus^@CA (around 7.8 ppm) became lower (as evidenced by the ratio of the peak height I1 of the hydroxyl group to the peak height I2 of the adjacent carbon-hydrogen peak) and splitting of the peak occurs (as observed in the magnified section in Figure S6B). In this case, hydrogen bonds may form between the hydroxyl in CA and the amino in the peptide. According to reverse-phase high-performance liquid chromatography experiments, the encapsulation rate of HBB^plus^CA was 72.8% (Figure S7). Moreover, no significant changes in HBB^plus^CA hydrodynamic size were observed in PBS containing 20% Fetal bovine serum (FBS) over 48 h (Figure S8). These results suggested that HBB^plus^CA displayed good stability and physicochemical properties. Thus, it deserves further exploration of its pharmacological properties.

### The HBB^plus^@CA has the ability to internalize cells, accumulate within metastatic tumors, and exhibit biosafety

**Figure 2:**
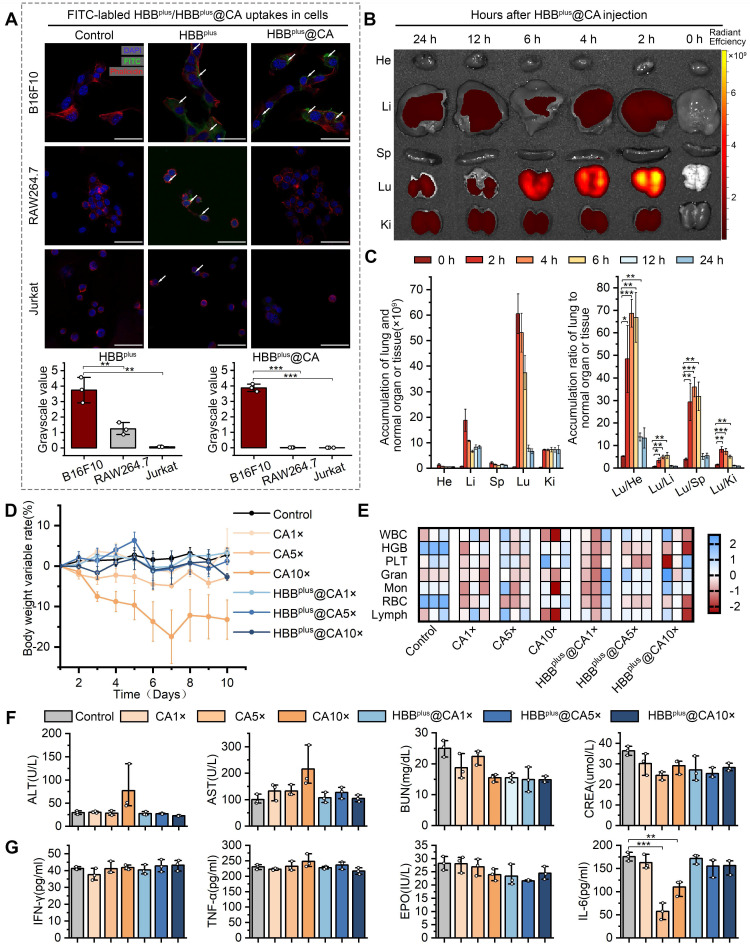
**HBB^plus^@CA Possessed Favorable biofunctions.** (A) Laser confocal microscopic images analysis of the cell internalization behavior of HBB^plus^/HBB^plus^@CA uptake in B16F10 cells, RAW 264.7 cells (B-C) *Ex vivo* fluorescent images (B) and analysis (C) of major organs from Cy5-labelled HBB^plus^@CA treated mice at 0 h, 2 h, 4 h, 6 h, 12 h, and 24 h post-injection. (D) Body weight changes of healthy C57BL/6 mice treated with CA or HBB^plus^@CA at different dosages. (E) Analysis of red blood cells (RBC), white blood cells (WBC), platelets (PLT), hemoglobin (HGB), granulocyte (GRAN), Monocytes (Mon), and lymphocytes (Lymph) in mice blood with the indicated treatments (n = 3/group). (F) Hepatotoxicity testing of the HBB^plus^@CA was measured by glutamic- oxalacetic transaminase (AST), glutamic-pyruvic transaminase (ALT), and renal toxicity testing measured by urea nitrogen (URN), creatinine (CREN). (G) Immunogenicity of HBB^plus^@CA in immune-competent C57BL/6 mice (n = 3/group) as measured by the concentration of tumor necrosis factor-α (TNF-α), Interferon γ (IFN-γ), erythropoietin (EPO) and interleukin-6 (IL-6) levels of peripheral blood. The data were presented as mean ± s.d. *, p < 0.05; **, p < 0.01; ***, p < 0.001.

The difficulty of penetrating the cell membrane and immune phagocytosis by T cells and/or macrophages severely restricted the clinical translation of peptide-derived therapeutics 46, 47, 51. Thus, we comparatively assessed the cellular internalization of HBB, HBB^plus^, and HBB^plus^@CA into B16F10 (metastatic melanoma) cells. To trace them, the HBB, HBB^plus,^ and HBB^plus^@CA were labeled to fluorescein isothiocyanate (FITC) in the N-terminal. Additionally, we stained the actin cytoskeleton of B16F10 cells with phalloidin-TRITC (red). As shown in Figure S9, HBB barely penetrated malignant melanoma cell membranes and entered the tumor cells. RGDSP optimization significantly improved the capabilities of HBB^plus^ for penetrating tumor cell membranes. Further, self-assembled nanoparticles HBB^plus^@CA exhibited significant internalization potential in tumor cells (Figure 2A). It's important to note that HBB^plus^@CA also significantly inhibited the phagocytosis of macrophages (RAW 264.7 cells) and T lymphocytes (Jurkat cells) more than HBB^plus^ (Figure 2A). These results indicated that the self-assembly supramolecular strategy achieved significant tumor enrichment properties and much longer circulation potential.

To validate the pharmacokinetics of HBB^plus^@CA, the fluorescence intensity of HBB^plus^@CA labeled Cy5-SE in the blood plasma extracted from healthy C57BL/6 mice was detected and quantified. As shown in Figure S10, HBB^plus^@CA exhibited a half-life of 6.10±2.10 h, indicating favorable *in vivo* blood circulation. Next, a classical lung metastasis of melanoma mouse model was used to explore further the *in vivo* distribution and tumor targeting of HBB^plus^@CA. As reported 52-55, B16F10 cells (5×10^5^ cells/mouse) were injected *via* the tail vein. To facilitate *in vivo* quantification, HBB, HBB^plus^, and HBB^plus^@CA were labeled with Cy5-SE fluorescent dye and administered intravenously to tumor-bearing C57BL/6 mice. The heart, liver, spleen, lung, and kidney were collected at different time points after injection, and the IVIS spectrum measured their fluorescence signals. The biodistribution of HBB^plus^, which contains RGDSP, and the peptide without RGDSP (HBB) in various organs was examined at 12 h and 24 h. As illustrated in Figure S11, HBB^plus^ exhibited superior tumor accumulation compared to HBB, indicating its effective targeting of tumors due to the presence of RGDSP. In this instance, HBB^plus^@CA exhibited a preferential accumulation in the lung tumor, indicating a favorable in vivo tumor targeting capability (Figures 2B&C).

Drug toxicity plays a critical role in the clinical translation of nanomedicines 56. Therefore, we have demonstrated the biological safety of the supramolecular peptide HBB^plus^@CA *in vivo*. We administered healthy C57BL/6 mice in seven groups: 1) PBS control, 2) HBB^plus^@CA 1× dose (4 mg/kg), 3) HBB^plus^@CA 5× doses (20 mg/kg), 4) HBB^plus^@CA 10× doses (40 mg/kg), 5) CA 1× dose (4 mg/kg), 6) CA 5× doses (20 mg/kg), 7) CA 10× doses (40 mg/kg), intraperitoneally, once every other day, six treatment cycles. According to body weight monitoring, mice receiving interventions in the CA 5× doses group and the 10× doses group achieved notable weight loss, especially in the 10× doses group. However, the HBB^plus^@CA 5× doses group and 10× doses group did not experience a significant decrease in body weight (Figure 2D). It appeared that HBB^plus^@CA had a better safety profile than CA, based on the above result. An additional biosafety assessment was conducted by performing routine blood and biochemistry tests, as well as a hematological evaluation. According to Figure 2E, neither HBB^plus^@CA nor CA affected red blood cells (RBC), white blood cells (WBC), platelets (PLT), hemoglobin (HGB), granulocytes (GRAN), monocytes (Mon) or lymphocytes (Lymph) in mice in comparison with controls. It was concluded from these studies that supramolecular peptides had no significant impact on hematopoiesis. Glutamic-pyruvic transaminase (ALT) and Glutamic-oxalacetic transaminase (AST) are key serological indicators for assessing liver function. The mice exposed to the CA 10× doses group displayed elevations of ALT and AST, whereas mice exposed to the HBB^plus^@CA 10× doses group did not demonstrate any significant changes in liver function (Figure 2F). According to the results of the renal function tests, HBB^plus^@CA and CA did not cause severe nephrotoxicity in mice (Figure 2F). Even with the high dose, HBB^plus^@CA and CA did not significantly increase tumor necrosis factor-α (TNF-α), interferon γ (IFN-γ), and erythropoietin (EPO) levels (Figure 2G). Notably, no significant elevation of IL-6 was seen in the HBB^plus^@CA group, even in the maximum-dose group. Pathological damage manifested in the organs of mice was further examined by hematoxylin-eosin (H&E) staining (Figure S12). We could see that the intervention of CA caused inflammatory cell infiltration in the lung tissue of mice in both the low-dose and high-dose groups. However, the lung tissue of mice receiving HBB^plus^@CA did not show any significant signs of inflammation (Figure S12). Moreover, no significant pathological changes were observed in the staining results of other organ tissues (heart, liver, spleen, and kidney) across all groups (Figure S12). Hence, based on our results, it is concluded that self-assembled nanoparticles HBB^plus^@CA are exceedingly safe and capable of avoiding adverse effects caused by CA, such as liver damage and lung inflammation.

### The HBB^plus^@CA suppressed MAPK and Wnt/β-catenin signaling pathways

**Figure 3:**
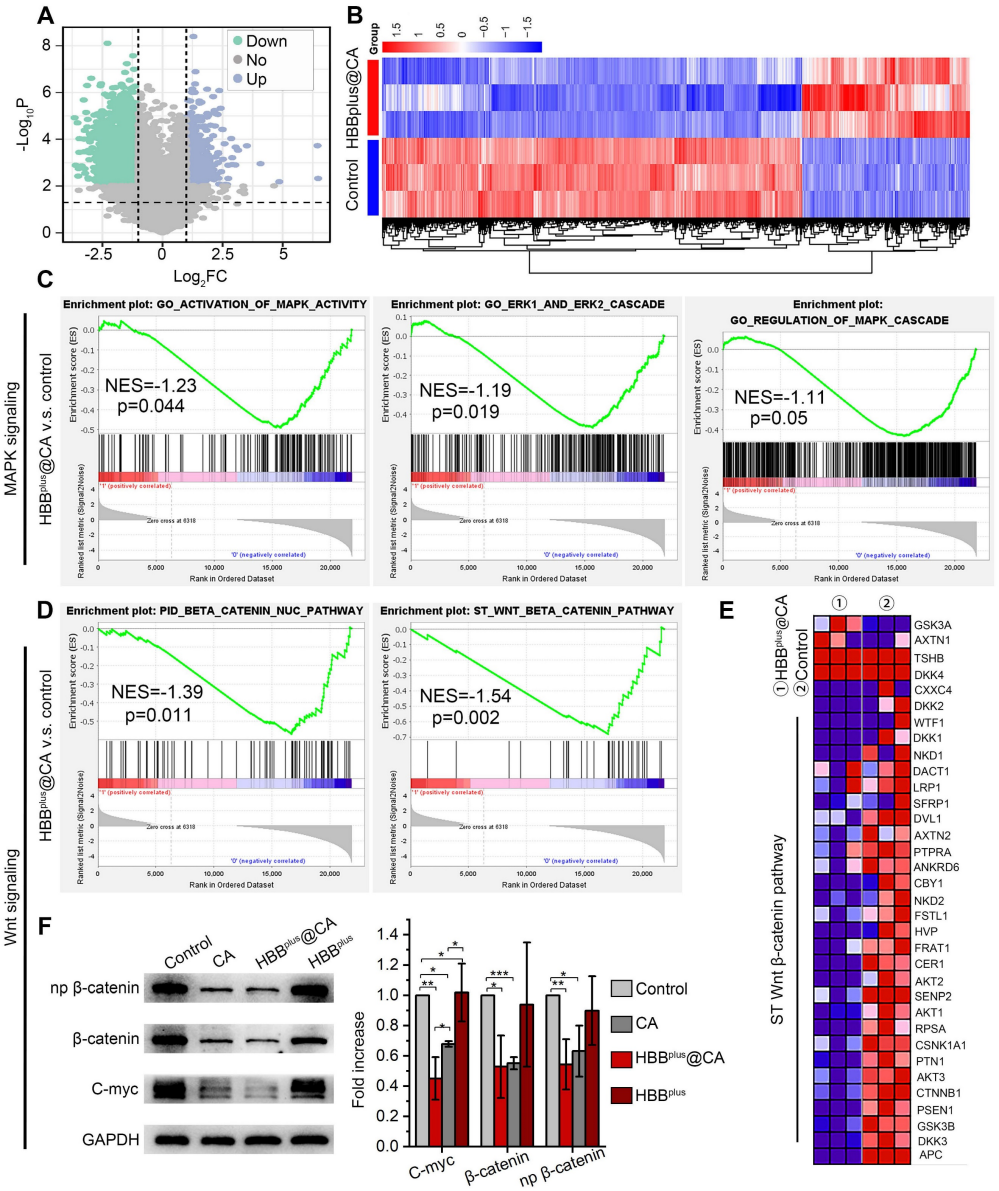
** HBB^plus^@CA nanoparticles suppressed the Wnt/β-catenin signal pathway *in vitro*.** Volcano plots of genes differentially expressed in B16F10 cells after exposure to 60μM HBB^plus^@CA for 24 h compared with mock-incubated cells (n = 3). (B) Heatmaps depict supervised clustering of the significantly modified genes between HBB^plus^@CA and control subgroups. (C) Gene set enrichment analysis (GSEA) of MAPK signaling in HBB^plus^@CA and control subgroups, involving activation of MAPK ability, ERK1/ERK2 cascade, and regulation of MAPK cascade pathway (n = 3 samples per group). NES, normalized enrichment score. (D) GSEA showing the Wnt/β-catenin signaling pathway and β-catenin nuc pathway differentially expressed in response to HBB^plus^@CA (n = 3). NES, normalized enrichment score. (E) A heatmap representing regulated genes by HBB^plus^@CA compared to control. (F) Western blot analysis for the expression of β-catenin, np-β-catenin, and C-myc proteins in B16F10 cells after different treatments. GAPDH was used as the loading control.

Next, we aimed to investigate further the underlying mechanisms and functional pathways of HBB^plus^@CA in anti-tumor. We attempted to quantify genomic alterations by comparing the transcriptome sequencing (RNA sequencing) of HBB^plus^@CA-treated and mock-treated cells. For this, B16F10 cells were inoculated in 6 well plates (2×10^5^/well), then inoculated with either HBB^plus^@CA (60 μM) or mock for 24 h. Next, cells were collected, and RNA was isolated for transcriptome analysis. As volcano plots in Figure 3A showed, HBB^plus^@CA triggered 2181 differential genes compared to mock treatment in B16F10 cells (Figure 3A). Subsequent clustering analysis also revealed that HBB^plus^@CA induced a noticeable gene change at the transcriptional level (Figure 3B). A gene set enrichment analysis (GSEA) revealed that the remarkably down-regulated pathways in HBB^plus^@CA-treated B16F10 cells involved activation of MAPK ability, ERK1/ERK2 cascade, and regulation of MAPK cascade pathway (Figure 3C). And the heat map also showed MAPK pathway inhibition by HBB^plus^@CA in melanoma cells (Figure S13-15). Besides, GSEA analyses confirmed that Wnt/β-catenin signaling pathway and β-catenin nuc pathway were pronounced down in B16F10 cells with HBB^plus^@CA treatment compared to mock-treated (Figure 3D). The heat map revealed similar results (Figure 3E, Figure S16). As a result, HBB^plus^@CA suppressed the MAPK/ERK and Wnt/β-catenin cellular pathways at the transcriptional level.

To unravel the alteration pathway at the protein level, B16F10 cells were inoculated with the treatments for 24 h. After that, the protein was purified for immunoblotting analysis. As expected, HBB^plus^@CA and HBB^plus^ significantly down-regulated the level of p-ERK1/2, which revealed its potential to inhibit the MAPK pathway (Figure S17, S18A). Meanwhile, HBB^plus^@CA significantly decreased the expression of the N-cadherin protein, a pivotal molecule in the epithelial-mesenchymal transition that is a classical pathway for tumor metastasis (Figure S17, S18B). As Figure 3F and S18C showed, CA and HBB^plus^@CA significantly down-regulated total β-catenin protein levels as well as the phosphorylation levels of β-catenin and its downstream gene c-Myc. Moreover, HBB^plus^@CA demonstrated more potent inhibition of the Wnt/β-catenin signaling pathway than CA. In summary, transcriptome sequences and immunoblotting data revealed HBB^plus^@CA-induced anti-tumor effects by downregulating the Wnt-β-catenin and MAPK/ERK pathways.

### The HBB^plus^@CA *in vitro* suppressed melanoma cell proliferation, migration, and invasion

**Figure 4:**
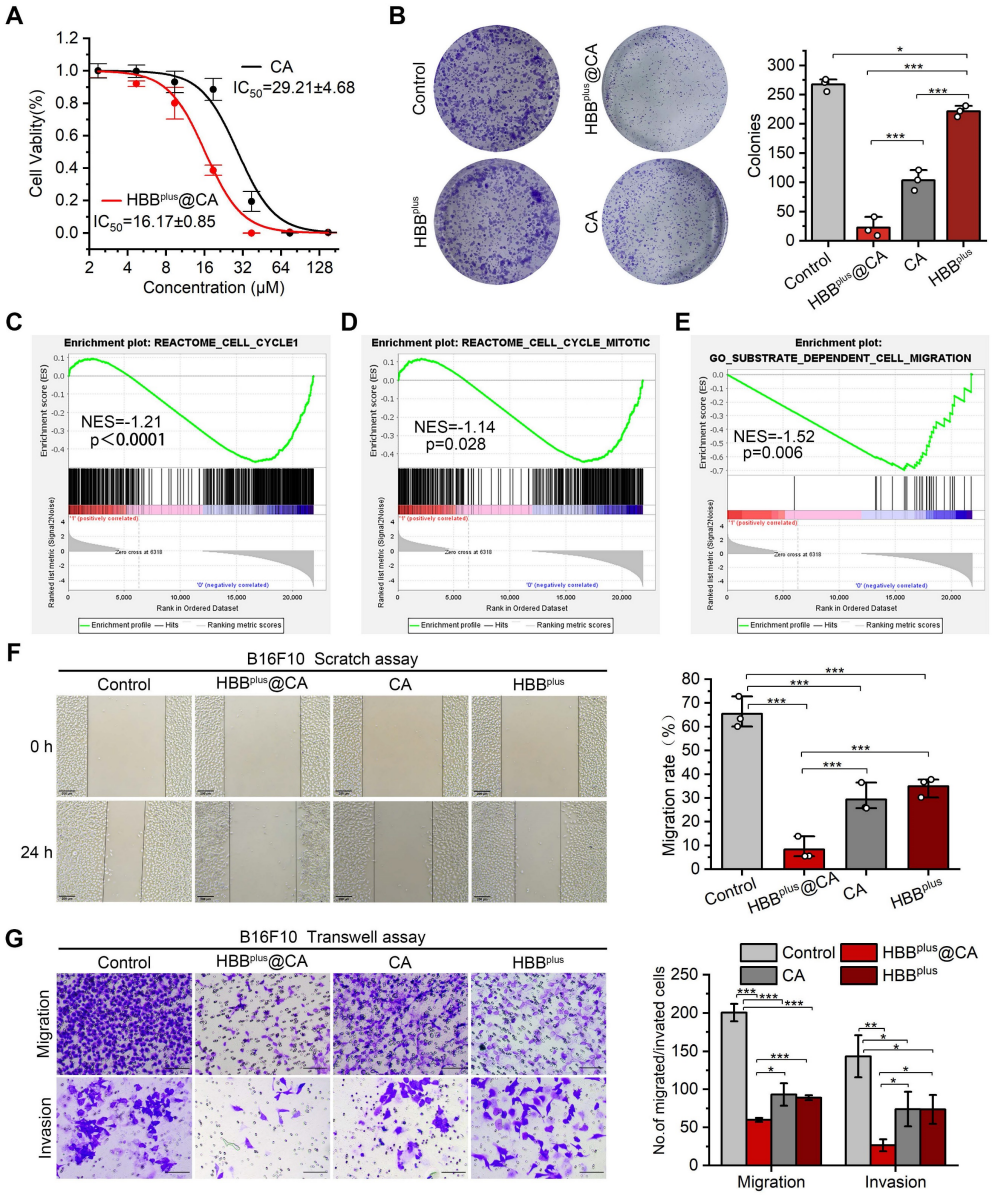
** HBB^plus^@CA *in vitro* inhibited tumor invasion and migration*.*** (A) Cell viability of B16F10 cells upon the HBB^plus^@CA or CA treatment. (B) Cell clone formation assay of B16F10 cells incubated with HBB^plus^@CA, HBB^plus^, or CA for 7 d. (C-E) GSEA analysis of HBB^plus^@CA -treated B16F10 cells compared to control cells involved in the Reactome cell cycle (C), Reactome cell cycle mitotic (D), and substrate-dependent cell migration (E) (n = 3). (F) Scratching experiments of B16F10 cells with HBB^plus^@CA, HBB^plus^, or CA drugs for 24 h (scale bar: 200 μm). (G) Transwell assays to monitor the effect of the drugs indicated on the migration and invasion of B16F10 cells (scale bar: 100 μm). The data were presented as mean ± s.d. *, p < 0.05; **, p < 0.01; ***, p < 0.001.

To explore the antitumor properties of HBB^plus^@CA at the cellular level, we measured the viability of B16F10 cells after treatment with HBB^plus^@CA. Firstly, as a negative control, the RGDSP-X peptide was synthesized from scrambled sequences of HBB and modified with RGDSP. As Figure S19A showed, RGDSP-X failed to inhibit the anti-tumor effect of B16F10 cells. In contrast, HBB^plus^ showed more potent antiproliferative activity in cytotoxicity assay (Figure S19A) and clone formation assay (Figure S19B). As well, HBB^plus^ also inhibited the migration of B16F10 cells in the scratch assay (Figure S19C) and the transwell migration and invasion assays (Figure S19D). The results above showed that HBB^plus^ exhibited good antitumor properties *in vitro*. Following that, we investigated the anti-tumor properties of self-assembled supramolecules HBB^plus^CA. According to Figure 4A, HBB^plus^@CA and CA exhibited concentration-dependent cytotoxicity to melanoma cells. Compared to the IC50 of CA, which was 29.21±4.68 μM, the IC50 of the supramolecular peptide HBB^plus^@CA was considerably lower, 16.17±0.85 μM. Hence, the supramolecular peptide significantly enhanced the antitumor efficacy of CA because of its bioactive molecules. The cell clone formation assay results showed that HBB^plus^@CA and CA significantly reduced the number of clones formed in B16F10 cells after 7 days of drug treatments compared to the control group (Figure 4B). Besides, HBB^plus^@CA has a stronger tumor suppressive effect than CA (Figure 4B). Flow cytometry was used to measure the impact of the indicated treatments on B16F10 cells. It was found that HBB^plus^CA and HBB^plus^ were significantly effective in increasing the apoptosis of tumor cells, particularly HBB^plus^CA. (Figure S20). Furthermore, using GSEA, we identified other gene sets associated with tumor proliferation and migration. Then, GSEA analysis and heat maps found that HBB^plus^@CA restrained the Reactome cell cycle (Figure 4C, Figure S21) and Reactome cell cycle mitotic pathways (Figure 4D, Figure S22). These results indicated that HBB^plus^@CA possessed high cytotoxicity against B16F10 melanoma cells. It should be noted that because of the controlled release of CA in HBB^plus^@CA, cell viability of HBB^plus^@CA was higher than that of free CA with a CA-equivalent dose of 30 μM.

As a next step, we tested the ability of supramolecular peptides to prevent tumor cell migration and invasion. GSEA analysis and heat map found that HBB^plus^@CA significantly inhibited the contrastive substrate-dependent cell migration activity of B16F10 melanoma cells (Figure 4E, Figure S23). Meanwhile, a scratch wound was harvested as part of the migration assay, and the healing process was observed. Based on a digital image, the areas of scratching without cells immediately following scratching and those of scratching without cells after the assay was calculated. It was shown in Figure 4F that, following 24 h of drug treatment, the migration rate of B16F10 cells was ~7 % in the HBB^plus^@CA group, ~27 % in the CA group, and ~36 % in the peptide group, indicating that the supramolecular peptide HBB^plus^@CA inhibited tumor cell migration more effectively than the control group. We observed the same results in the transwell migration assay. Furthermore, tumor cells' invasive abilities play a critical role in metastasis. Using a transwell invasion assay (Figure 4G), we assessed the ability of HBB^plus^@CA to inhibit tumor cell invasion. Additionally, HBB^plus^@CA was superior to both CA and HBB^plus^ in blocking B16F10 cell invasion from the upper chamber to the lower section. Together, these results demonstrated that HBB^plus^@CA inhibited tumor cells' proliferative and invasive effects *in vitro*.

### The HBB^plus^@CA inhibited pulmonary metastasis of melanoma *in vivo*

**Figure 5:**
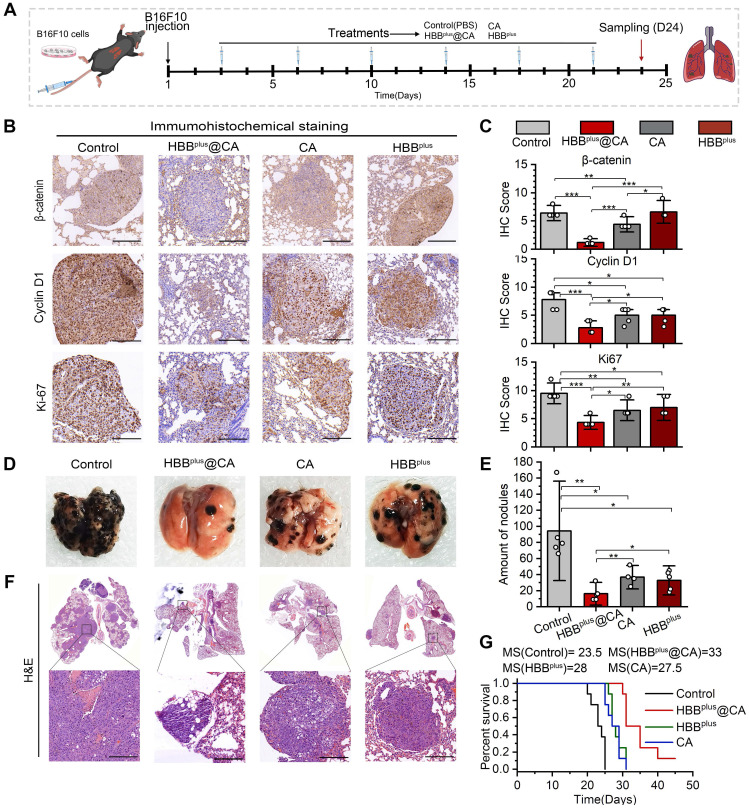
** HBB^plus^@CA *in vivo* suppressed the pulmonary metastasis of melanoma.** (A) The flow of mouse melanoma lung metastasis transplantation tumor model establishment. (B) The IHC staining of Ki-67, β-catenin, and Cyclin D1 in tumor sections from mice with the indicated treatments (scale bar: 200 μm). (C) The Ki-67, β-catenin, and Cyclin D1 relative expression levels in mice tumor sites with the indicated treatments (n =5). (D) The photographs of the tumor-bearing lung with the indicated treatments. (E) Statistical chart of lung nodules in the indicated groups (n =5). (F) H&E staining of the tumor-bearing lung with the indicated treatments (scale bar: 200 μm. (G) Survival curves for mice with melanoma lung metastasis after the indicated treatments (n =5). The data were presented as mean ± s.d. *, p < 0.05; **, p < 0.01; ***, p < 0.001.

On account of the anticancer efficacy of the supramolecular peptide *in vitro*, we were motivated to investigate their performances *in vivo*. As mentioned before, the mouse model of pulmonary metastasis of melanoma was used to simulate the metastatic process of melanoma through blood flow, with 5 × 10^5^ B16F10 cells injected intravenously into C57BL/6 mice. There were four groups (n = 5 per group) of mice: 1) PBS control, 2) HBB^plus^@CA (4 mg/kg), 3) CA (4 mg/kg), and 4) HBB^plus^ (4 mg/kg). A six-cycle treatment was performed two days after the cell injection, as indicated in Figure 5A. On day 24, after tumor cell inoculation, tumor proliferation and nodule number in the lungs were assessed in mice.

A histological examination of the tumor was performed to examine the antitumor mechanisms involved with the above treatments (Figures 5A and B). HBB^plus^@CA therapy significantly inhibited the proliferative capacity of tumors, as demonstrated in Figure 5B&C, since Ki-67 expression was lower in the HBB^plus^@CA group (Scored 3.8) compared to the control group (Scored 8.3), the HBB^plus^ group (Scored 6.2), and the CA group (Scored 5.7). At the same time, the β-catenin expression (Figure 5B) and the np β-catenin expression (Figure S24) of the melanoma cells were significantly reduced in response to HBB^plus^@CA. Additionally, downstream pathway proteins of the Wnt/β-catenin, Cyclin D1 (Figure 5B), and c-Myc (Figure S24) were also considerably reduced in the HBB^plus^@CA group, which suggested that HBB^plus^@CA may block the activation of Wnt/β-catenin. We also found a remarkably reduced level in the expression of p-ERK1/2 in the tumor cells after the therapy of HBB^plus^@CA (Figure S24). Notably, the HBB^plus^@CA significantly modulated the critical proteins expression of tumor metastasis, inhibited N-cadherin, and dramatically increased E-cadherin (Figure S24). As a result of these immunohistochemical analyses, HBB^plus^@CA could inhibit the Wnt-β-catenin and MAPK/ERK signaling pathways to exert anti-tumor effects and inhibition of tumor metastasis. Obviously, the trend of lung metastasis suppression was also found in photo images (Figure 5D). As for the lung tumor nodules (Figure 5E), the mean value of metastatic lung foci in the PBS group was 94 ± 41 per lung. The average number of metastatic foci dropped to 32 ± 12 and 37 ± 10 per lung for the HBB^plus^ and CA groups, respectively, while for the HBB^plus^@CA group, it was only 16 ± 9 per lung (Figure 5F). HBB^plus^@CA also significantly improved the median survival time from 23.5 days (control group) to 33 days for mice with melanoma lung metastases (Figure 5G). Furthermore, we compared the antitumor effects of HBB^plus^CA with the combination of HBB^plus^ and CA and found that HBB^plus^CA was significantly more effective than the combo therapy group at battling lung metastases (Figure S25). By monitoring the rate of body weight changes (Figure S26) during treatment and the H&E staining of primary organs in mice post-treatment, we did not find any significant toxicity events of HBB^plus^@CA (Figure S27). The results of this study indicated that HBB^plus^@CA had the most excellent anti-metastatic effect compared to other treatment groups. In short, the supramolecular peptide strategy for targeting intracellular proteins will offer new effective, and safe options for treating tumor metastasis.

## Conclusions

In summary, using an RGD-modified MAPK/ERK inhibitor (HBB^plus^) and a small molecule catenin inhibitor (CA), we developed a bifunctional supramolecular peptide, HBB^plus^@CA, which was self-assembled into nanospheres primarily through hydrophobic and hydrogen bonding interaction. As modified by the RGD peptide, HBB^plus^CA was significantly internalized in malignant melanoma cells and greatly enriched in the tumor-bearing lung tissue of mice. In addition, HBB^plus^@CA exhibited a good circulation time and was biologically safe. As a result, HBB^plus^@CA inhibited melanoma cell proliferation, migration, and invasion by simultaneously inhibiting MAPK/ERK and Wnt/β-catenin signaling pathways, providing more excellent anti-tumor activity than HBB^plus^ or CA alone or combined treatment. Further, the HBB^plus^@CA was highly effective at inhibiting lung metastasis and significantly extending survival in mice with metastatic melanoma B16F10. In short, we developed a realistic strategy for inhibiting tumor metastasis and advanced the development of these technologies for efficient and precise cancer therapy.

## Supplementary Material

Supplementary figures and methods.
