# Supplementary material for: A de novo dual-targeting supramolecular self-assembly peptide against pulmonary metastasis of melanoma
Source: Theranostics. 2023 Jun 26;13(11):3844–55. doi: 10.7150/thno.83819 (PMC10334834; doi:10.7150/thno.83819)
Supplement: Supplementary file 1 — Supplementary figures and methods. [file thnov13p3844s1.pdf]

Supplementary

A *de novo* dual-targeting supramolecular self-assembly peptide against pulmonary metastasis of melanoma

Jingjing Wang[1, †], Xiaoqiang Zheng[1, 2,†], Xiao Fu[1,†], Aimin Jiang[1],
Yu Yao[1], Wangxiao He [1,2,3,*]

1. Department of Medical Oncology, The First Affiliated Hospital of Xi'an Jiaotong University, Xi'an 710061, China.
2. Institute for Stem Cell & Regenerative Medicine, The Second Affiliated Hospital of Xi'an Jiaotong University, Xi'an 710004, China.
3. Department of Talent Highland, The First Affiliated Hospital of Xi'an Jiao Tong University, Xi'an 710061, China.

† These authors contributed equally.

* Corresponding authors:
Email: hewangxiao5366@xjtu.edu.cn (W. He)

Supplemental Figures.

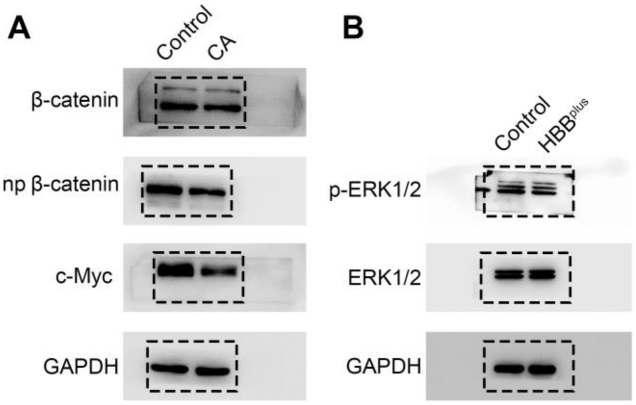

Figure S1. Origin western blot images of proteins in B16F10 cells after treatments.

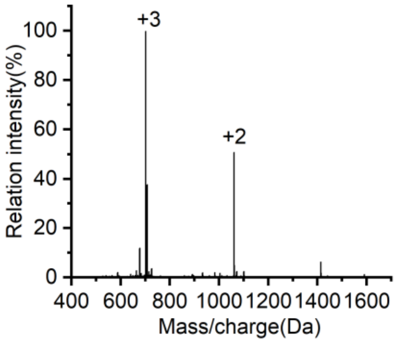

Figure S2. Electrospray ionization-mass spectrometry (ESI-MS) of HBB^{plus}.

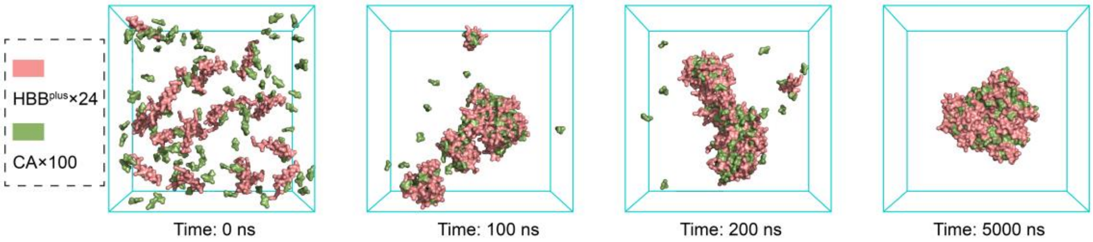

Figure S3. Simulation of HBB^{plus}@CA molecular dynamics self-assembly (The mass ratio of HBB^{plus} and CA is 1:1).

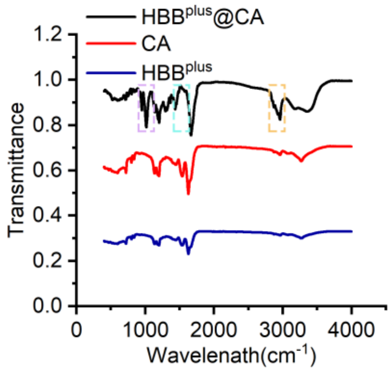

Figure S4. Fourier transform infrared spectroscopy (FT-IR) of HBB^{plus}@CA, CA, and HBB^{plus}.

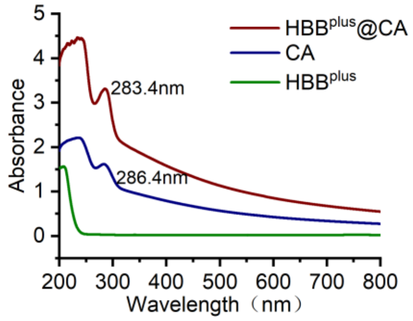

Figure S5. Ultraviolet-visible spectra of HBB^{plus}@CA, CA, and HBB^{plus} in H_2O at a concentration of 0.3 mg/ml.

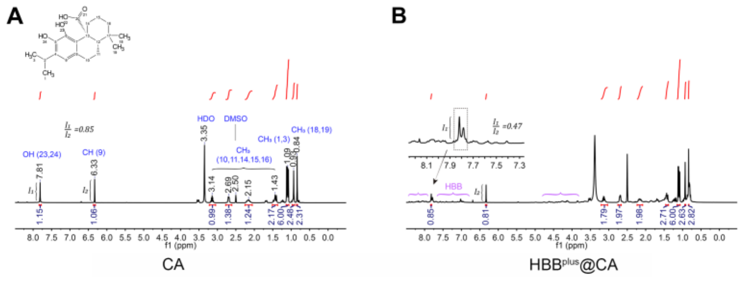

Figure S6. ^{1}H NMR spectrum of HBB[plus] (D_2O as a solvent), CA (DMSO-d6 as a solvent) and HBB[plus]@CA (D_2O and DMSO-d6 as solvent), 400 MHz.

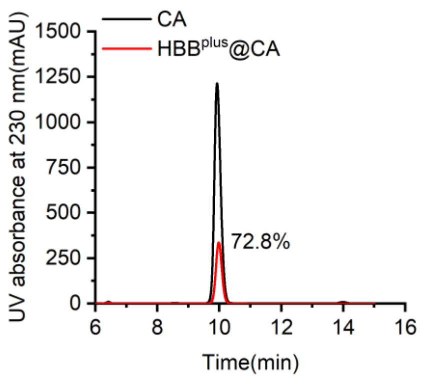

Figure S7. Encapsulation efficiency of HBB^{plus}@CA using reverse-phase high-performance liquid chromatography (CA concentration 1 mg/ml; HBB^{plus}@CA concentration 1mg/ml, centrifugation at 10000 rpm for 10 min)).

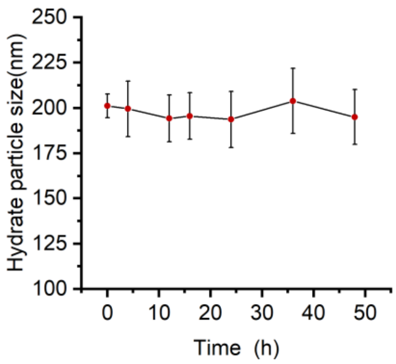

Figure S8. Size stability of HBB^{plus}@CA in PBS containing 20% FBS.

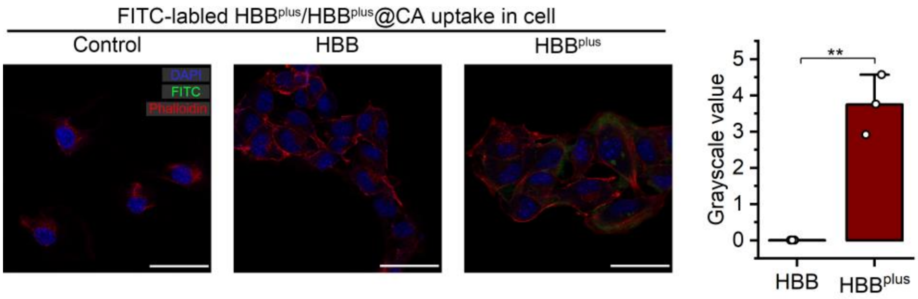

Figure S9. Microscopic images of the cell internalization behavior of HBB^{plus}/HBB uptake in B16F10 cells (scale bar: 50 μm), Image J analysis of HBB/HBB^{plus} uptake in B16F10 cells, The data were presented as mean ± s.d. *, $p < 0.05$; **, $p < 0.01$; ***, $p < 0.001$.

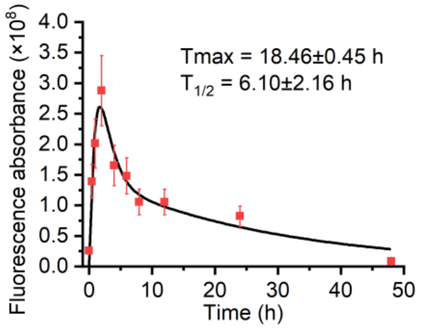

Figure S10. A microplate reader detected and quantified the pharmacokinetics of HBB^{plus}@CA labeled Cy5-SE in the blood plasma extracted from healthy C57BL/6 female mice (excitation: 649 nm, emission: 670 nm).

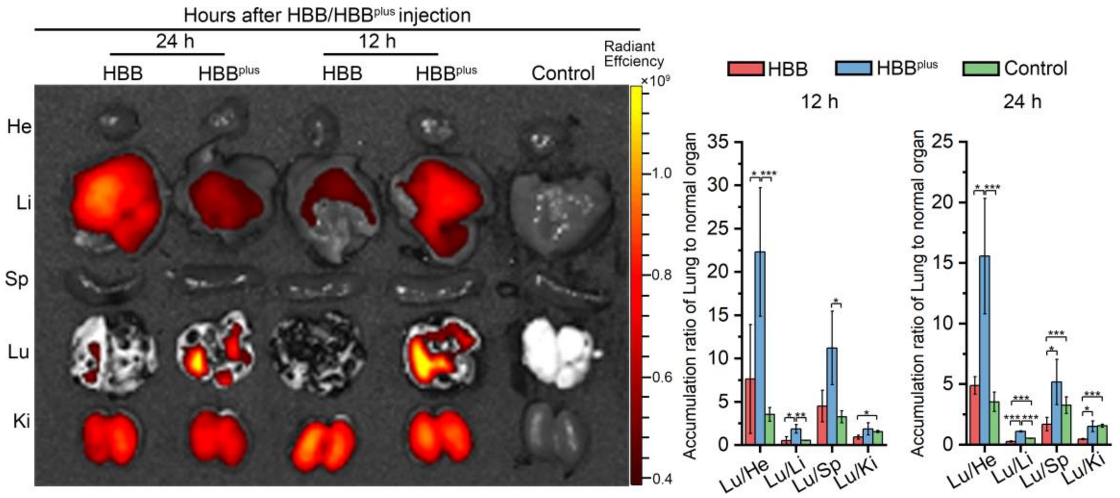

Figure S11. Ex vivo fluorescent images and analysis of major organs from Cy5-SE labeled HBB^{plus}/HBB treated C57BL/6 mice tumor-bearing pulmonary metastatic melanoma at 0 h, 12 h, and 24 h post-injection. He, heart; Li, liver; Sp, spleen; Lu, lung; Ki, kidney. The data were presented as mean ± s.d. *, $p < 0.05$; **, $p < 0.01$; ***, p <

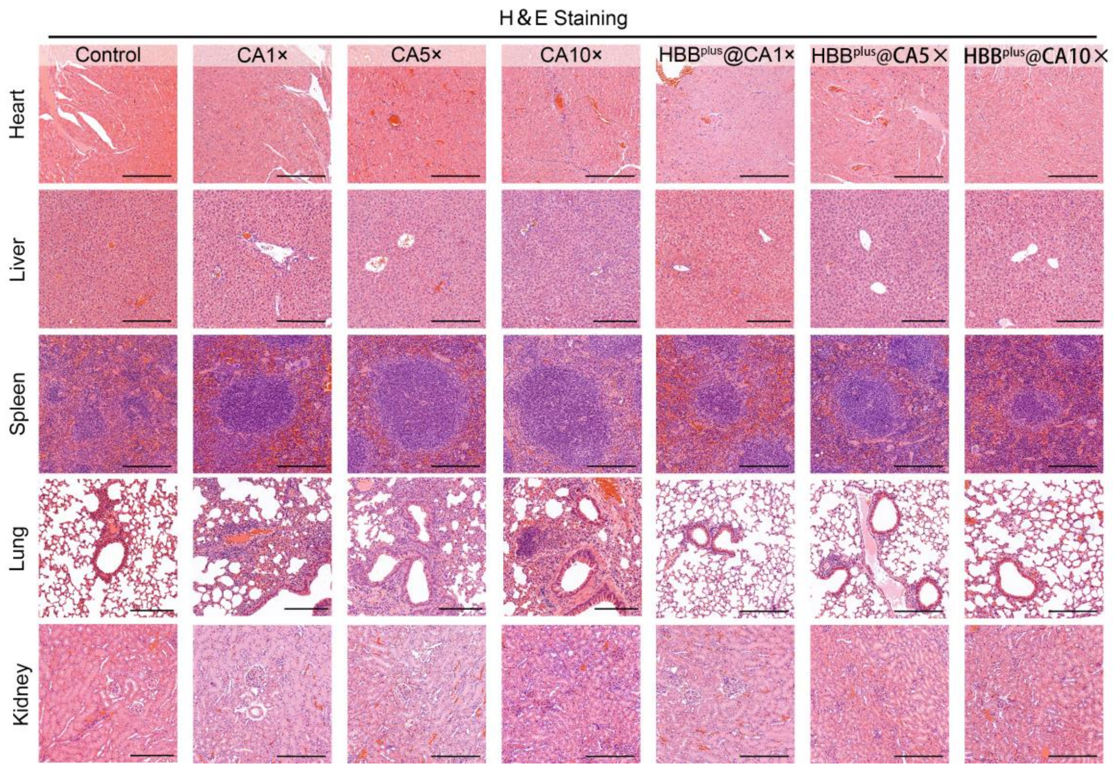

Figure S12. H&E staining of heart, liver, spleen, lung, and kidney tissues in healthy C57BL/6 female mice with the treatment: CA and HBB^{plus}@CA were set in different dose groups from low to high: 1× dose (4 mg/kg), 5× doses (20 mg/kg), 10× doses (40 mg/kg), (scale bar: 200 μm).

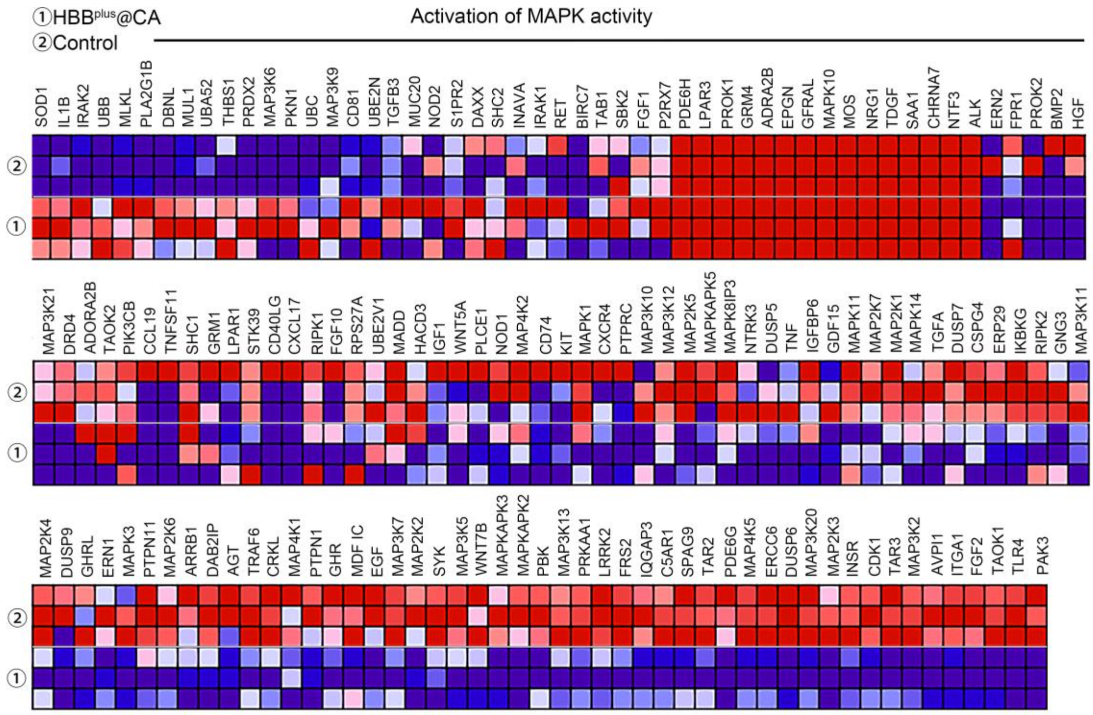

Figure S13. Heatmap of genes regulation in activation MAPK activity.

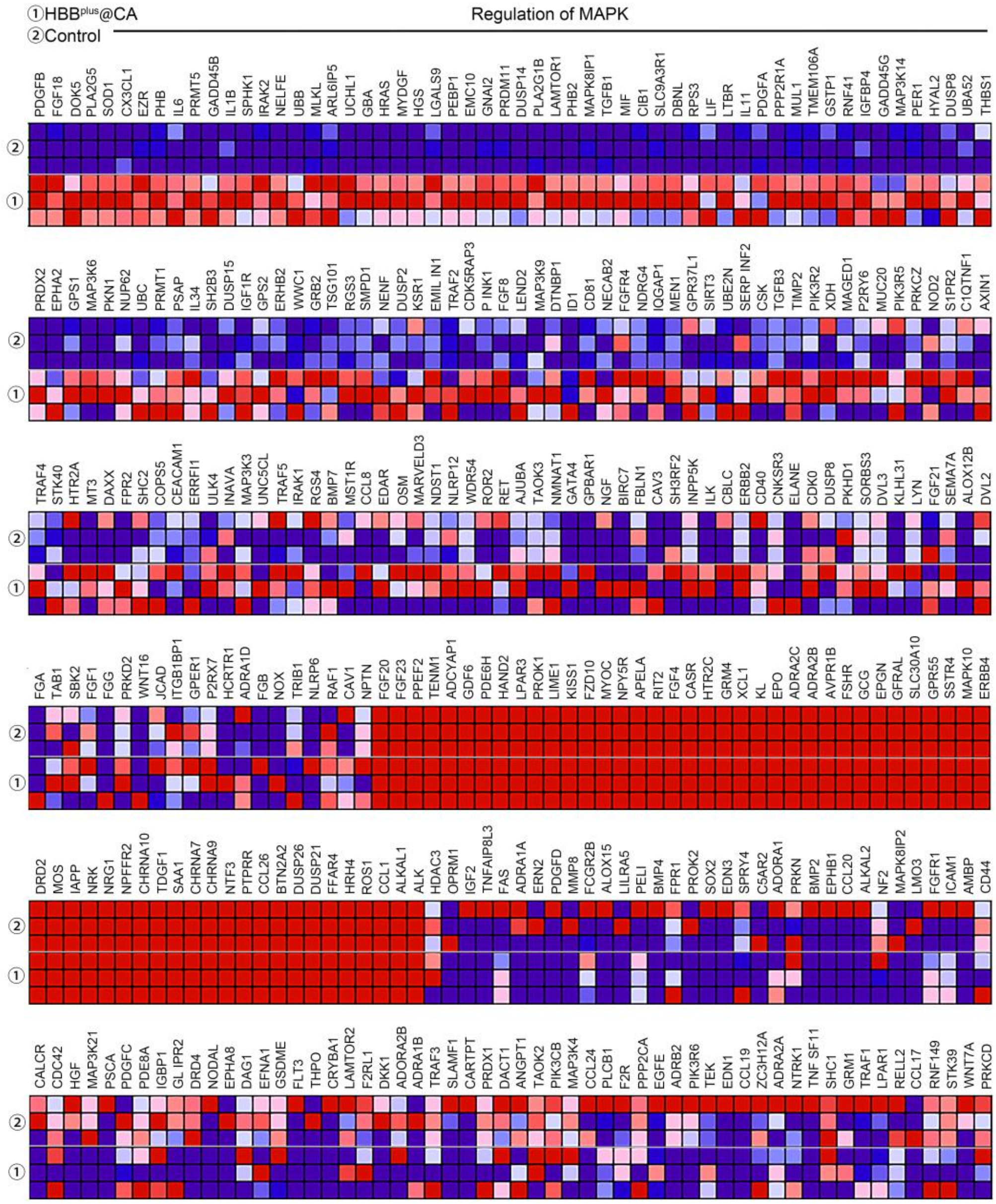

Figure S14. Heatmap of genes changes in the regulation of MAPK signaling pathway.

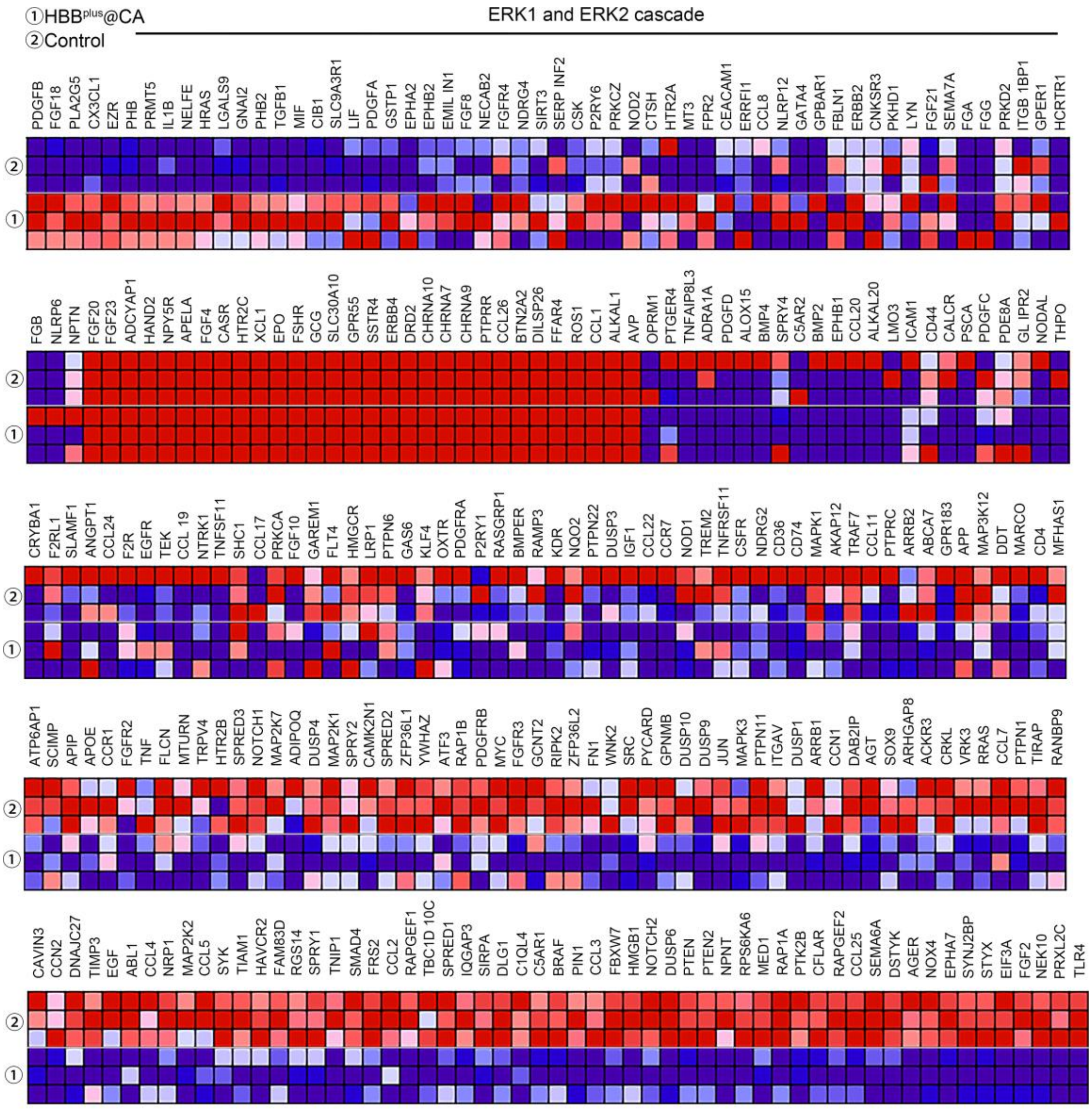

Figure S15. Heatmap of gene changes in the regulation of ERK1/2.

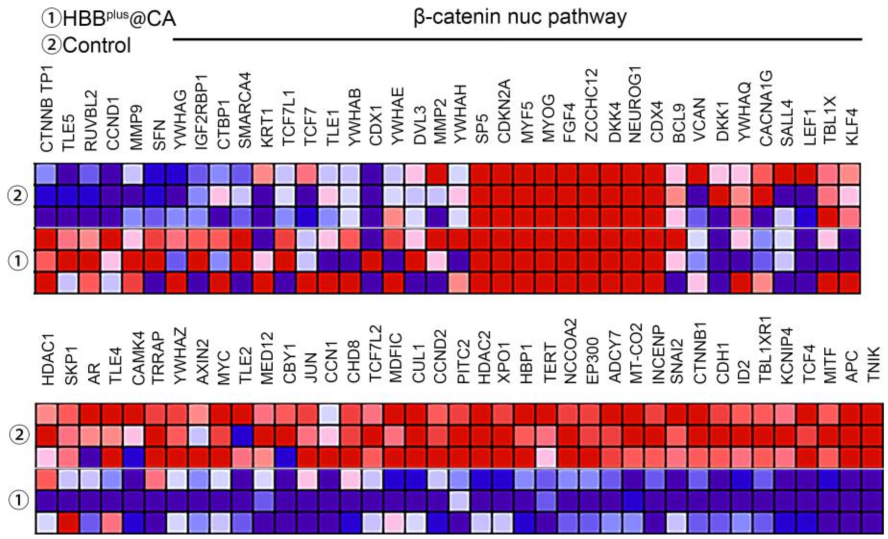

Figure S16. Heatmap of genes regulation in β-catenin nuc signaling pathway.

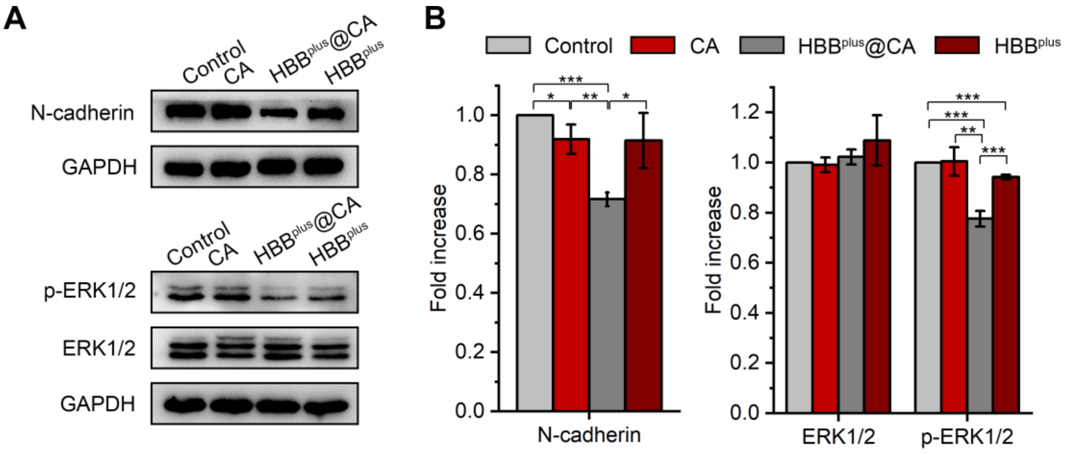

Figure S17. Western blot analysis for the level of N-cadherin, p-ERK1/2, and ERK1/2 proteins in B16F10 cells after different treatments. GAPDH was used as the loading control. The data were presented as mean ± s.d. *, $p < 0.05$; **, $p < 0.01$; ***, $p < 0.001$.

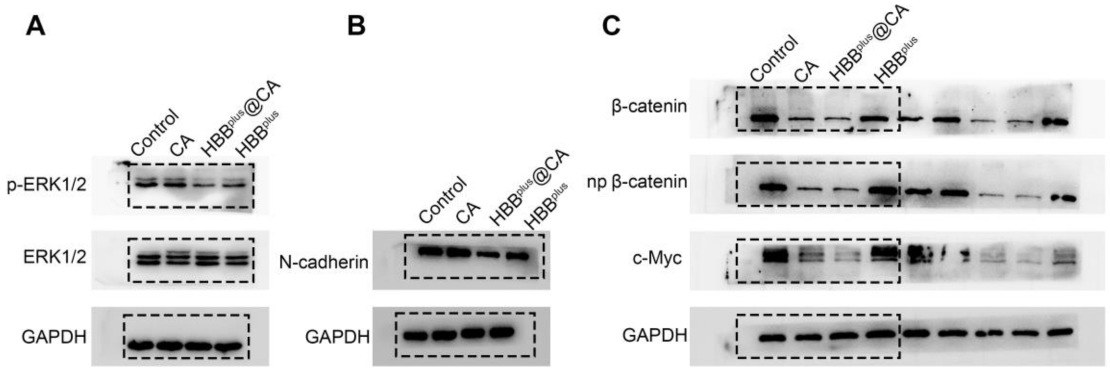

Figure S18. Origin western blot images of proteins in B16F10 cells after treatments.

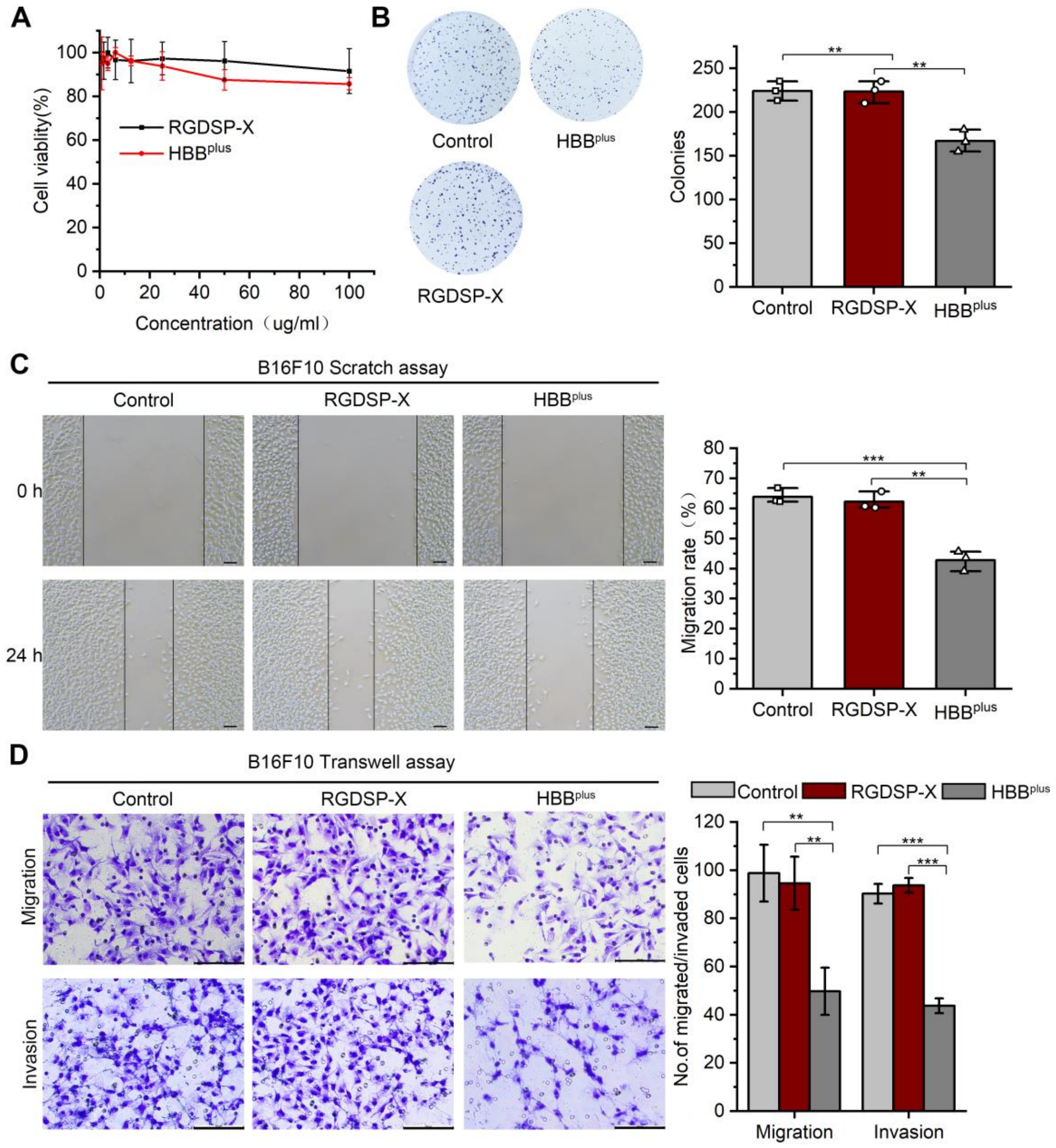

Figure S19. The HBB^{plus} and RGDSP-X in vitro suppressed melanoma cell proliferation, migration, and invasion. (A) Cell viability of B16F10 cells upon the HBB^{plus} or RGDSP-X treatment. (B) Cell clone formation assay of B16F10 cells incubated with HBB^{plus} or RGDSP-X for 7 days. (C) Scratching experiments of B16F10 cells with HBB^{plus} or RGDSP-X drugs for 24 h (scale bar: 100 μm). (D) Transwell assays to monitor the drugs' effect on the migration and invasion of B16F10 cells (scale bar: 100 μm). The data were presented as mean ± s.d. *, $p < 0.05$; **, $p < 0.01$; ***, $p < 0.001$.

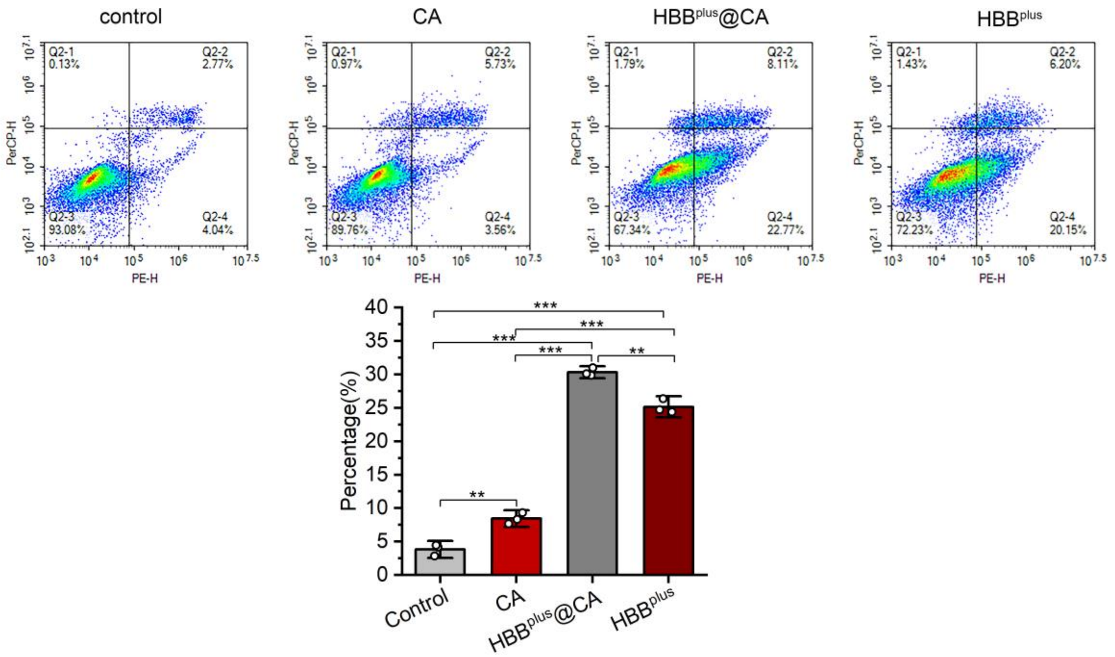

Figure S20. Cell apoptosis of B16F10 treated by HBB^{plus}@CA, CA, HBB^{plus}, and Control. The data were presented as mean ± s.d. *, $p < 0.05$; **, $p < 0.01$; ***, $p < 0.001$.

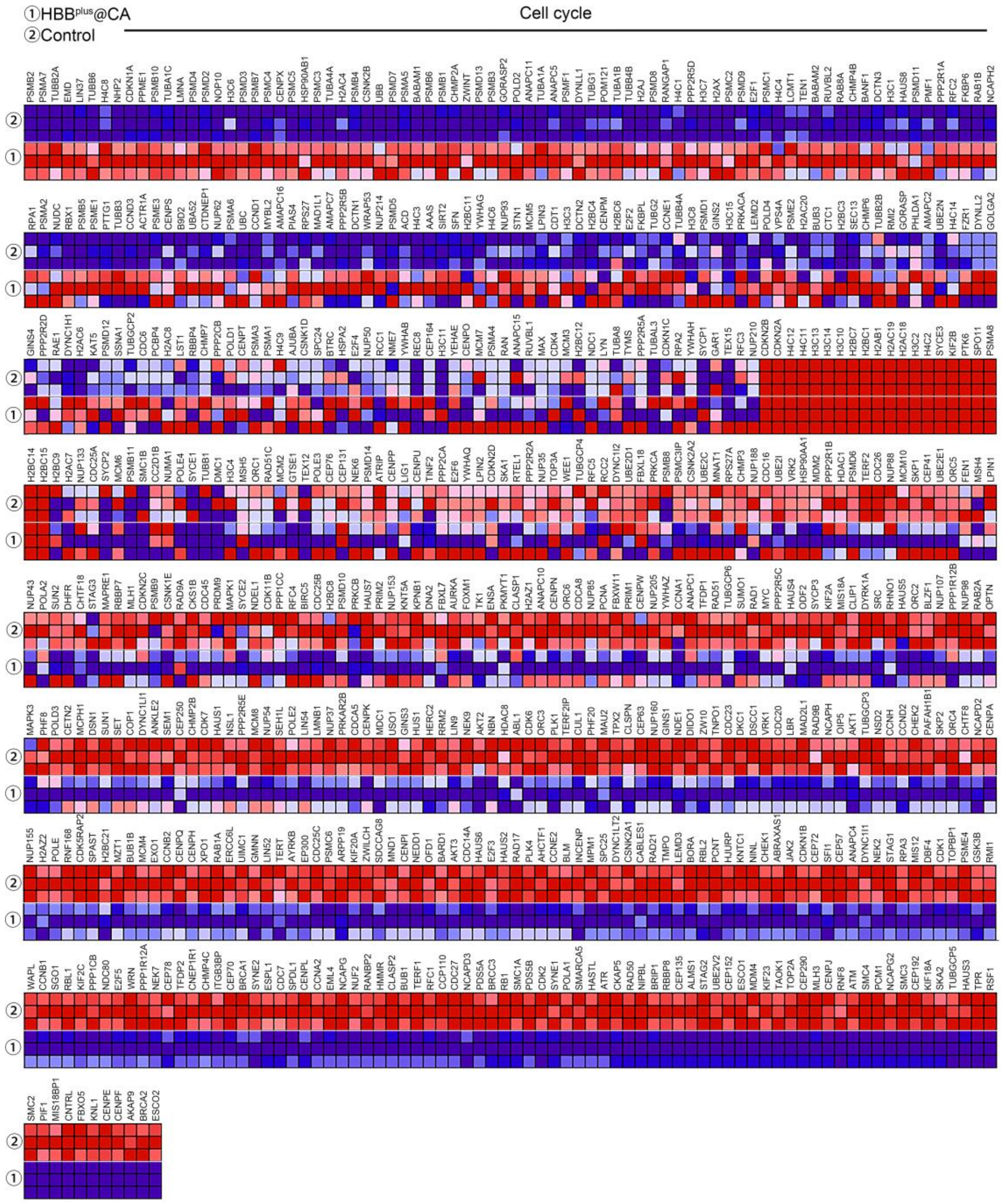

Figure S21. Heatmap of genes regulation in the cell cycle of B16F10.

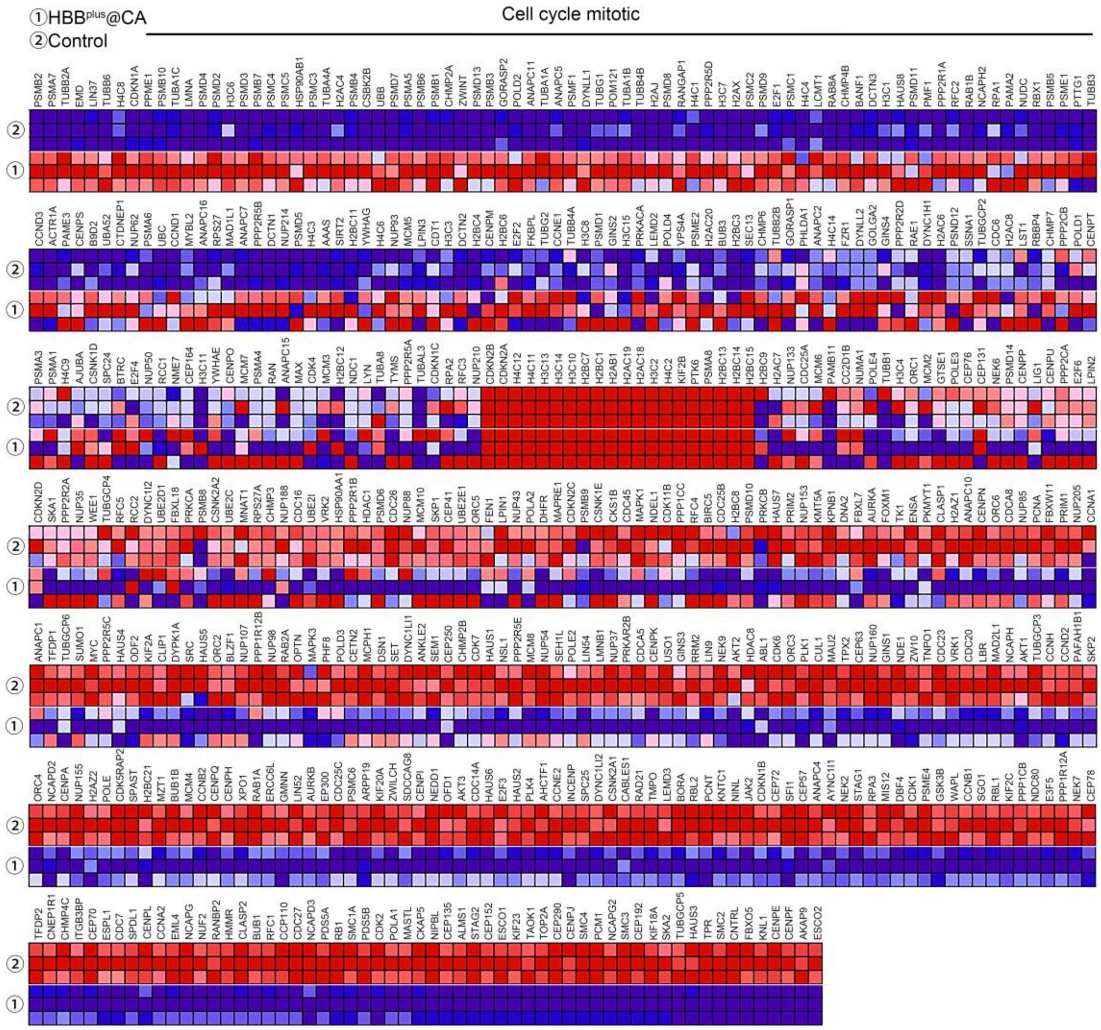

Figure S22. Heatmap of genes regulation in cell cycle mitotic of B16F10.

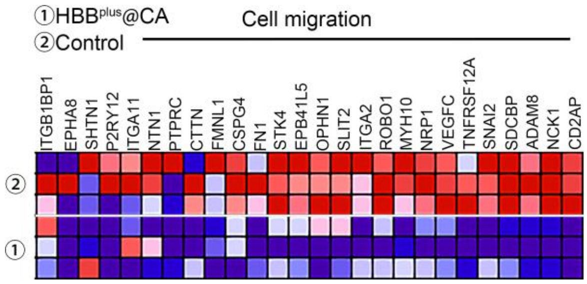

Figure S23. Heatmap of genes regulation in cell migration of B16F10.

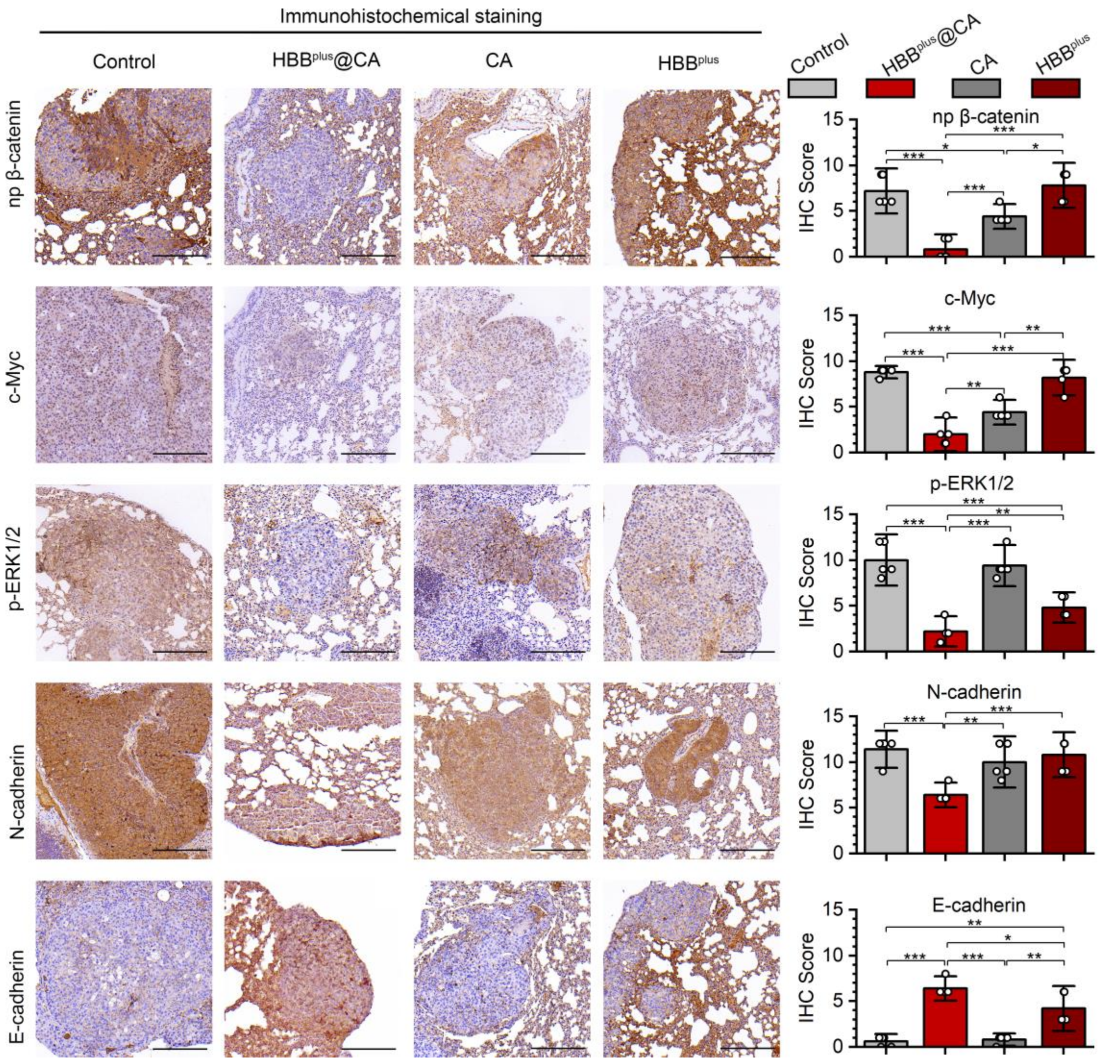

Figure S24. The IHC staining of np β-catenin, c-Myc, p-ERK1/2, N-cadherin, and E-cadherin in tumor sections from mice with the indicated treatments (scale bar: 200 μm). The data were presented as mean ± s.d. *, $p < 0.05$; **, $p < 0.01$; ***, $p < 0.001$.

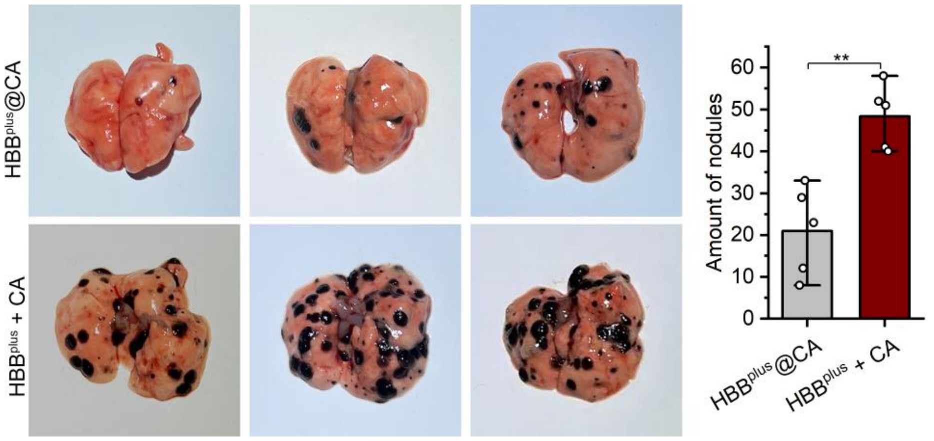

Figure S25. The photographs of the tumor-bearing lung with the indicated treatments: HBB^{plus} + CA, HBB^{plus}@CA. Statistical chart of lung nodules in the indicated groups (n =5). The data were presented as mean ± s.d. *, $p < 0.05$; **, $p < 0.01$; ***, $p < 0.001$.

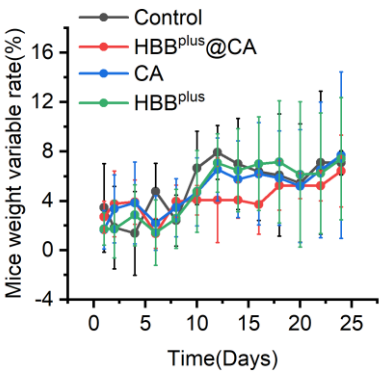

Figure S26. Body weight of healthy C57BL/6 mice treated with CA (4 mg/kg), HBB^{plus} (4 mg/kg), HBB^{plus}@CA (4 mg/kg), and PBS.

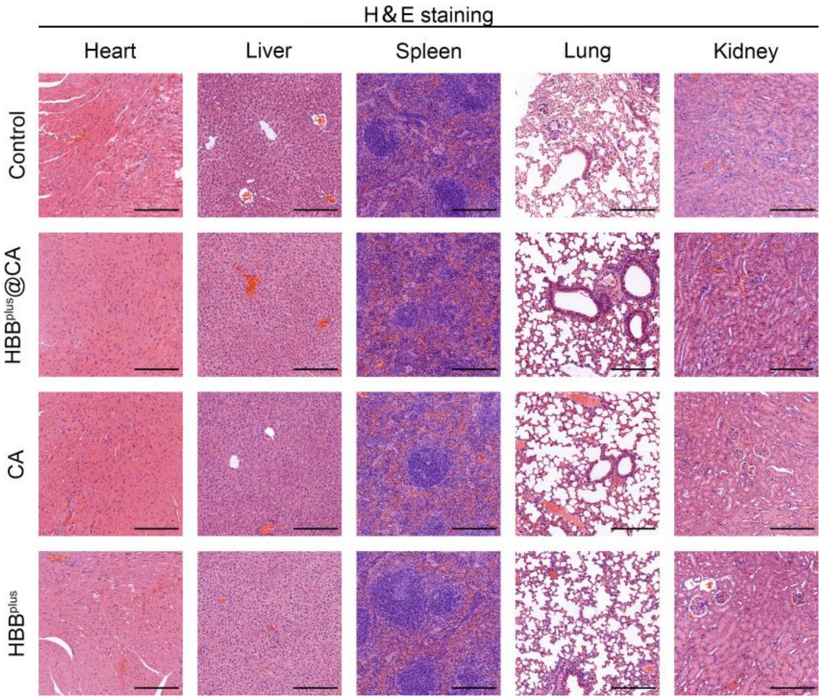

Figure S27. H&E staining of heart, liver, spleen, lung, and kidney tissues in C57BL/6 mice tumor-bearing pulmonary metastatic melanoma female mice with the treatment: CA (4 mg/kg), HBB^{plus} (4 mg/kg), HBB^{plus}@CA (4 mg/kg) and PBS, (scale bar: 200 μm).

Materials and methods

Bioinformatics data acquisition and processing

GSE19234 series was obtained from the GEO dataset for further correlation analysis and survival analysis, with the GPL570 platform being used to annotate the gene symbol. The duplicated genes were replaced with mean values, and the raw expression matrix was log-transformed. Survival analysis was performed *via* R software, "survminer" and "survival" packages. The "surv_cutpoint" function determined the cut-off value of MAPK15 and CTNNB1 and therefore transformed them into categorical variables. Convert it to the high and low expression group to draw the KM curve. Use the gg-scatter function in the ggpubr package to visualize the correlation between MAPK15 and CTNNB1.

Synthesis of HBB^{plus} and HBB^{plus}@CA

HBB^{plus} peptides were synthesized on appropriate resins on a CS bio 336X automated peptide synthesizer using the optimized HBTU activation/DIEA in situ neutralization protocol developed by an HBTU/HOBt protocol for Fmoc-chemistry SPPS. After cleavage and deprotection in a reagent cocktail containing 88% TFA, 5% phenol, 5% H_2O, and 2% TIPS, crude products were precipitated with cold ether and purified to homogeneity by preparative C18 reversed-phase HPLC. The molecular masses were ascertained by electrospray ionization mass spectrometry (ESI-MS).

The HBB^{plus} were dispersed in distilled water at a concentration of 1 mg/mL; CA (Energy Chemical, China) was dissolved in DMSO at a concentration of 1mg/10 uL and then dropwise added into the HBB^{plus} solution at the mass rate of 4:1, 2:1, 1:1, 0.5:1, 0.25:1, and 30 min ultrasonic concussion.

Synthesis of HBB and RGDSP-X

HBB (ENFRLLGNVLVCVLA) and RGDSP-X (ANVLNECVPVGRLLL, a control peptide X of a scrambled amino acid sequence designated scrambled-HBB, were chemically synthesized.) sources were obtained from QYAOBIO (Shanghai) Ltd.

Characterization of HBB^{plus}@CA

We combined the GROMACS 2018.3 software package with the MARTINI coarse-grained model (version 2.2) to conduct dynamics simulations on a system composed of 24 peptide molecules and

100 CA molecules in an aqueous solution for 5000 ns.

The morphology structure was observed on Lorenz transmission electron microscopy (TEM), performed on a Talos F200X. EDS elemental mappings were acquired using an FEI Talos F200x STEM and JEOL-ARM (200 F) 200 kV FEG-STEM. The hydrodynamic size distribution (1 mg/mL in H_2O, 1 mL) was obtained from the dynamic light scattering (DLS) measurement (Malvern Zetasizer Nano ZS system). The surface chemical structure of modified nanocrystals was evaluated by Fourier transform infrared (FT-IR) spectroscopy (Nicolet 6700) and UV−vis absorption spectra (Shimadzu 3000 spectrophotometer). The structural identification of modified nanocrystals was evaluated by a 400 MHz JEOL Nuclear Magnetic Resonance Spectrometer (NMR).

Cell culture

Mouse melanoma cell line B16F10, mouse macrophage cell line RAW 264.7, and human leukemic T-cell line Jurkat were obtained from the Chinese Academy of Science Cell Bank (Shanghai, China). B16F10 and RAW 264.7 cells were maintained in DMEM medium, Jurkat was cultured in RPMI-1640 medium, and the above mediums were supplemented with 10% FBS, 100 U/mL penicillin, and 100 μg/mL streptomycin. All cells were maintained at 37°C in an atmosphere of 5% CO_2.

Cell viability analysis

Melanoma cells B16F10 were inoculated uniformly in 96 empty plates according to 2000 cells. After 24 h, different gradient concentrations of HBB^{plus}@CA or CA, HBB, or RGDSP-X were intervened at 37°C in an atmosphere of 5% CO_2 for 72 h. The *in vitro* cell viability was measured using a standard Alamar blue (Thermo Fisher Scientific, China) assay in the B16F10 cells.

Transwell assays

B16F10 cells were planted in 24-well plates at a density of 1×10^5 cells per well. Cells were put into the upper chamber with or without a Matrigel-coated membrane (1:7 dilution, Corning). B16F10 cells were planted in 24-well plates at a density of 1×10^5 cells per well. After incubation of CA, HBB^{plus}, HBB^{plus}@CA, RGDSP-X with a concentration of 30 μM into the lower chamber containing 15% FBS medium for 24h -48h. Cells that migrated or invaded across the membrane were fixed with 4% PFA for 20 min and then stained with 0.1% crystal violet solution for 20 min. The chamber was repeatedly cleaned with distilled water and captured under a light microscope

(Leica).

Scratch wound healing assay

B16F10 cells at 5 × 10^5 cells/well were inoculated into the 6-well plate at 37 °C with 5% CO_2 for 24 h. After the cells formed a confluent monolayer, a scratch was created in the center of the monolayer with a pipette tip (100 μL). The monolayer membrane was washed with PBS three times, and then 60 μM of CA， HBB^{plus}, RGDSP-X, or HBB^{plus}@CA containing 1% fetal bovine serum was added to each well. The images of cells invading the scratch were captured at indicated time points (0 h, 24 h) with a Leica microscope (Longbase, China), and the pictures were analyzed independently with Image J. All samples were assayed in triplicate.

Cell apoptosis analysis.

Cell apoptosis was evaluated by flow cytometric analysis using the FITC Annexin V Apoptosis Detection Kit (BD Falco, China). Briefly, cells were treated with HBB^{plus}， HBB^{plus}@CA, and CA for 24 h at a concentration of 40 μM. Cells were harvested, washed twice with cold PBS, and re-suspended in 1 × binding buffer at a concentration of 1 × 10^6 cells/mL Cells (1 × 10^5 cells/100 μL) were transferred to a centrifugal tube, followed by adding 5 μL of FITC Annexin V and 5 μL of PI. After gentle vortexing and a 15 min incubation in the dark at room temperature, 400 μL of 1× binding buffer was added to the tube, and cells were analyzed by FACS.

Cellular uptake analysis

3 × 10^4 B16F10 cells/well, 3 × 10^5 RAW264.7 cells/well, and 3 × 10^5 Jurkat cells/well were seeded into Nunc Glass Bottom Dish at 37 °C with 5% CO_2 for 24 h. FITC-labeled HBB, HBB^{plus}, and HBB^{plus}CA were incubated with cells for 6 h at a concentration of 30 μM, respectively. After the medium was removed, PBS was used to wash it three times, and 4% formaldehyde was used to fix it for 10 min. 0.2% Triton X-100 destroyed the cell membrane for 10 min. DAPI (Sigma-Aldrich, USA) was used to label the cell nuclei for 5 min. Finally, a super-resolution confocal microscope was used to observe the cell uptake situation.

Transcriptome analyses

B16F10 cells were seeded in 6-well plates and left to cultivate overnight. Then cells were treated with HBB^{plus} and HBB^{plus}@CA, respectively, at a concentration of 60 μM for 24 h. And RNA was

isolated using the TRIzol reagent (Ambion, USA). RNA sequencing libraries were constructed using the NEBNext® Ultra RNA Library Prep Kit for Illumina® (NEB England BioLabs). Fragmented and randomly primed 2 × 150 bp paired-end libraries were sequenced using Illumina HiSeq X Ten. Heat maps and Gene Expression Enrichment Analysis were generated using the Qlucore Omics Explorer 3.2. Pathway analysis was performed using Ingenuity Pathway Analysis (IPA) software.

Western blot analysis

B16F10 Cells were treated with the indicated treatments respectively. Total protein from cells was extracted using RIPA lysis buffer containing protease inhibitors, and equal amounts of protein lysates were separated by 10% SDS-PAGE, transferred onto PVDF membranes, and probed using primary and secondary antibodies. The primary antibodies were as follows: anti-β-catenin (Abcam, USA; 1: 5000), anti-np β-catenin (Cell Signaling Technology, USA; 1: 1000), anti-p-ERK1/2 (Proteintech, USA; 1: 1000); anti-ERK1/2 (Cell Signaling Technology, USA; 1: 1000); anti- C-myc (Abcam, USA; 1: 1000), anti-N-cadherin (Cell Signaling Technology, USA; 1: 1000) and anti-GAPDH (Proteintech, USA; 1: 5000).

Mouse study

All mice were purchased from the Laboratory Animal Center of Xi'an Jiaotong University. Animals were housed under standard specific pathogen-free conditions with standard chow and typical light/dark cycles. All experimental procedures involving animals were conducted in accordance with Institution Guidelines and were approved by the Laboratory Animal Center of Xi'an Jiaotong University (approval number: 2021-1735).

Quantification of HBB^{plus}@CA pharmacokinetics

HBB^{plus}@CA labeled with Cy5-SE (MedChemExpress, USA) was injected intravenously at 200 μL (1 mg/mL) into healthy female C57BL/6 mice. The main organs were obtained at 0 h, 0.5 h,1 h, 2 h, 4 h, 6 h, 12 h, 24 h, and 48 h after injection. The mice were euthanized, and blood plasma extracted from the mice was detected and quantified by a microplate reader (excitation: 649 nm, emission: 670 nm); the fluorescence absorbance was analyzed using Origin. All samples were assayed in triplicate.

***In vivo* Fluorescence Imaging**

HBB^{plus}@CA labeled with Cy5-SE was injected intravenously at 200 μL (1 mg/mL) into C57BL/6 mice with tumor-bearing pulmonary metastatic melanoma. The main organs were obtained at 0h, 2 h, 4 h, 6 h, 12 h, and 24 h after injection and were used for detection in the CRI Maestro imaging system *in vivo*. HBB^{plus} and HBB labeled with Cy5-SE fluorescent dye were injected intravenously at 200 μL (1 mg/mL) into C57BL/6 mice with tumor-bearing pulmonary metastatic melanoma. The main organs were obtained at 12 h and 24 h after injection and were used for detection in the CRI Maestro imaging system *in vivo*. All samples were assayed in triplicate.

Construct a lung metastasis model of melanoma

The lung metastasis model of melanoma was constructed as follows: B16F10 cells (5×10^5 cells per 100 μL) were transplanted into healthy C57BL/6 mice by intravenous injection at 5-6 weeks of age. After 3 days, the mice were randomly divided into the PBS control group, CA group (4 mg/kg), HBB^{plus} group (4 mg/kg), HBB^{plus}@CA group (4 mg/kg), HBB^{plus} + CA group (4 mg/kg) tail-vein injected, every other day, summed six cycles. The experiment was terminated, and the mice were euthanized. For histological examination, the tumor, liver, kidney, heart, spleen, and lung tissues were fixed with formaldehyde, dehydrated, sliced into 4 μm sections, and subjected to H&E or immunohistochemical staining.

H&E and immunohistochemistry (IHC) staining

Tumor-bearing lungs and primary organ tissues were dissected and immersed in formalin solution. Then they were paraffin-embedded, sectioned with a 4 μm thick. Nextly, the sections were stained with hematoxylin-eosin (H&E) according to standard histopathological techniques. Besides, under conventional immunohistochemical staining, the expression of Ki-67 (Proteintech, USA; 1: 400), β-catenin (Thermo Fisher, China; 1: 2000), np β-catenin (Cell Signaling Technology, USA; 1: 100), Cyclin D1 (Cell Signaling Technology, USA; 1: 1000), c-Myc (Abcam, USA; 1: 200), p-ERK1/2 (Proteintech, USA; 1: 300); N-cadherin (Proteintech, USA; 1: 500), E-cadherin (Proteintech, USA; 1: 500) in tumor-bearing lungs sections were detected. Tumor-bearing lung sections were scanned by Pannoramic DESK and quantified.

To evaluate immunostaining intensity (I), we used a numeric score ranging from 0 to 3, reflecting

the intensity: 0, no staining; 1, weak staining; 2, moderate staining; and 3, intense staining. To evaluate immunostaining area (A), we used a numeric score ranging from 1 to 4, reflecting the intensity: 1, positive area 90%. Using an Excel spreadsheet, the mean score was obtained by multiplying the intensity score (I) by the percentage of the positive area, and the results were added together (total score: I × A).

Acute toxicity test *in vivo*

Healthy female C57BL/6 mice of 5-6 weeks were selected and treated by tail vein injection. CA and HBB^{plus}@CA were set in 3 dose groups from low to high, 1× dose (4 mg/kg), 5× doses (20 mg/kg), 10× doses (40 mg/kg), every other day for 2 weeks, and then the experiment was terminated. The effects of the drugs on the physiological indexes of the mice, such as body weight, appetite, locomotion, and mental health, were observed. The mice were tested for blood and biochemical routine, and to evaluate the biosafety of HBB^{plus}@CA by physical signs, hematology, and pathology, H&E staining was performed on the important organs of the mice.

ELISA assays

Enzyme-linked immunosorbent assays (ELISAs) were performed with paired antibody sets, as recommended by the manufacturer (NeoBioscience, Shenzhen, PRC; ml bio, Shanghai, PRC).

Statistical analysis

Statistical analyses were performed using a two-sided Student's t-test. $p < 0.05$ was considered significant. Data were expressed as mean ± s.d. or s.e. The Kaplan-Meier curves were used to depict the survival time. Outcomes of the statistical analyses were presented using the symbols: *, $p<0.05$; **, $p<0.01$; ***, $p<0.001$.
